# A new *Epseptimavirus* bacteriophage vB_SalS-SIY1lw as a potential antimicrobial alternative to multidrug-resistant *Salmonella* Infantis

**DOI:** 10.1038/s41598-025-31311-8

**Published:** 2026-02-05

**Authors:** Yen-Te Liao, Angela Voelker, Abigail R. Arellano, Yujie Zhang, Leslie A. Harden, Alexandra Salvador, Vivian C. H. Wu

**Affiliations:** Produce Safety and Microbiology Research Unit, Department of Agriculture, Agricultural Research Service, Western Regional Research Center, Albany, CA 94710 USA

**Keywords:** *Salmonella* infantis, Multidrug-resistant, Lytic phage, *Epseptimavirus* genus, Microbiology, Bacteriophages

## Abstract

*Salmonella* Infantis is an emerging pathogen highly associated with antibiotic-resistant issues and has contributed to increasing foodborne illness in recent years. Thus, finding novel antimicrobial agents is urgent for the solution. The objective was to characterize a newly isolated *Epseptimavirus* phage with antimicrobial activity against multidrug-resistant *S.* Infantis strains. *Salmonella* phage vB_SalS-SIY1lw (or SIY1lw) is a new member of the phages belonging to the *Epseptimavirus* genus. SIY1lw contained the receptor binding protein (ORF 23) and tail fiber protein (ORF 43) genes—both associated with bacterial host recognition and binding—similar to that in *Salmonella* phage OSY-STA (the *Epseptimavirus* genus) and *Escherichia* phage DaisyDussoix (the *Tequintavirus* genus), respectively. For biological traits, SIY1lw has a latent period of 30 min and an estimated burst size of 42 PFU/CFU. The phage was polyvalent against *S.* *enterica* (Infantis and Newport) and non-pathogenic *E. coli* strains. The in vitro antimicrobial activity test showed that the phage with MOI of 1,000 is the most effective in reducing *S.* Infantis FSIS7823 and FSIS4921 by 1 and 0.8 log, respectively, over the 6-h treatment at 25 °C. These findings indicate that the new *Epseptimavirus* phage SIY1lw has future potential to develop an antimicrobial alternative to multidrug-resistant *S.* Infantis strains.

**Supplementary Information:**

The online version contains supplementary material available at 10.1038/s41598-025-31311-8.

## Introduction

Non-typhoid *Salmonella enterica*, a zoonotic disease commonly inhabited in the gastrointestinal tract of broilers, is one of the most frequently occurring foodborne pathogens in the United States; the group of pathogens can cause more than 1 million cases of Salmonellosis every year, including 26,500 hospitalizations and 420 deaths^1,2^. Among these serovars, *S.* Infantis has emerged as one of the most predominant *Salmonella* isolates in the United States since 2016^1,3^. Mejía et al. found that *S*. Infantis contributed more than 95% and 50% of the *Salmonella* serovars isolated from poultry-associated and human samples, respectively^4^. *S.* Infantis became so prevalent primarily due to antimicrobial resistance, high persistence to environmental stresses, genetic adaptivity, and emerging variants^5,6^; among which, REPJFX01 was the most widespread and persistent variant involved in various outbreaks in the United States and other parts of the world^7^. An increasing number of *S.* Infantis strains isolated from poultry and human origins contains the plasmid of emerging S. Infantis (pESI), which renders these strains resistant to multiple antibiotics^7–9^. The pESI plasmid is likely transferred to other *Salmonella* serovars, such as Senftenberg, Muenchen, and Agona, even though the potential transfer mechanism is not confirmed^10^. Most importantly, the strains with pESI-like mega-plasmid have enhanced bacterial fitness against environmental stresses, such as resistance to disinfectants and increased biofilm-forming ability, thus posing a significant threat to public health and substantial economic loss for the poultry industry^5^.

Bacteriophages (or phages) are bacterial viruses ubiquitous in the ecosystem, among which lytic phages are frequently studied as alternative antimicrobial agents to enhance the efficacy of existing intervention technologies in the food industry^11,12^. Additionally, the host-specific feature of a lytic phage primarily depends on its binding to specific receptor proteins on the bacterial outer membrane and enables promising activity against antibiotic-resistant bacteria^13,14^. In a previous study, multiple *Salmonella*-infecting phages isolated from various water sample sources showed antimicrobial activity against different *Salmonella* serovars, including antibiotic-resistant strains^15^. Sevilla-Navarro et al. used lytic phages following chemical sanitation to significantly reduce *S.* Infantis to non-detectable levels at broiler farms, better than utilizing only chemical sanitizers^9^. In favor of developing effective hurdle antimicrobial interventions or phage cocktails to combat constantly changing bacterial pathogens^6^, discovering new suitable phages with antimicrobial potential is needed. Therefore, the objective was to characterize a newly isolated *Epseptimavirus* phage with antimicrobial activity against multidrug-resistant *Salmonella* Infantis strains.

## Results

### Biological characterization

#### Phage morphology and growth factor

**Fig. 1:**
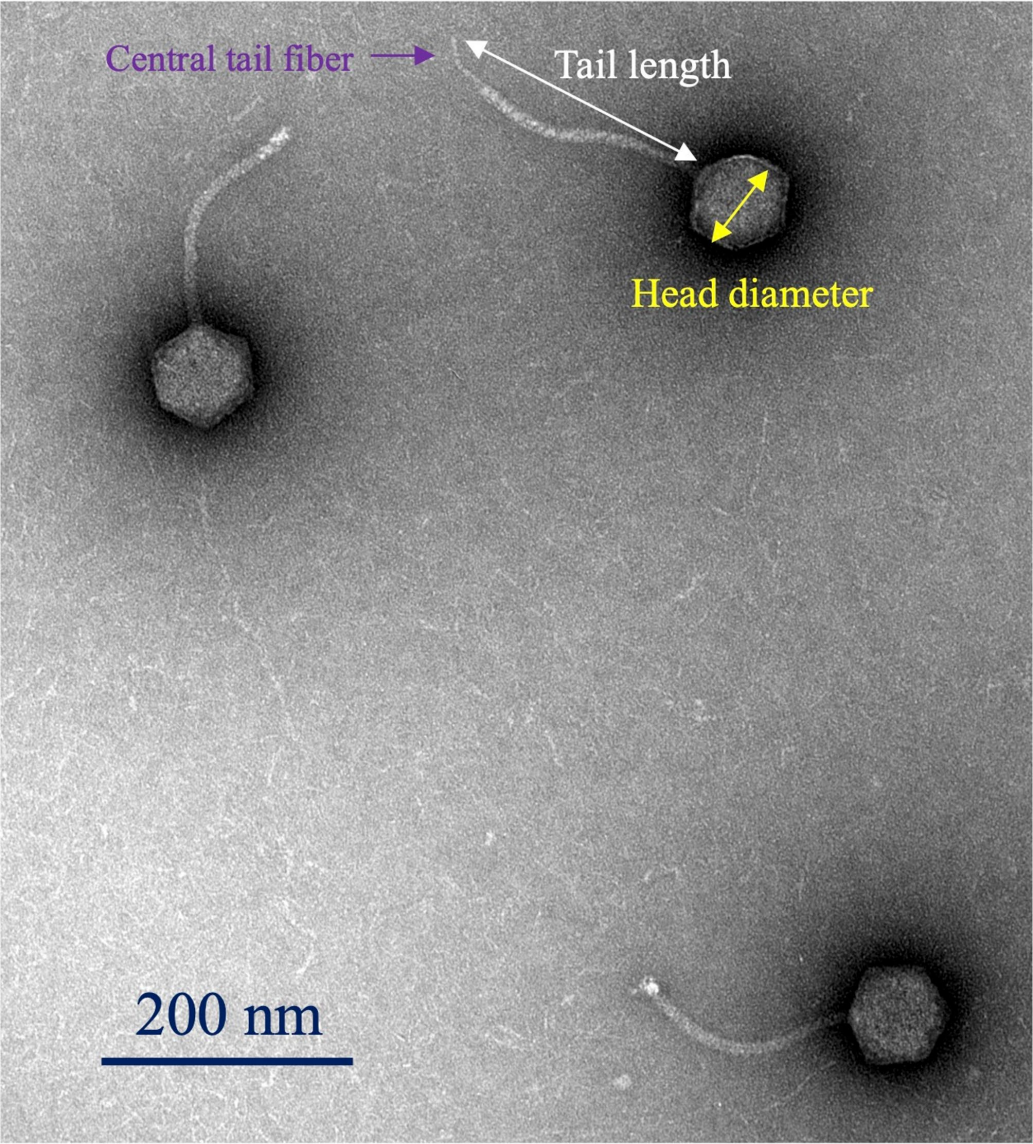
Transmission electron microscopy image of phage SIY1lw, with central tail fiber at the end of the tail structure.

**Fig. 2:**
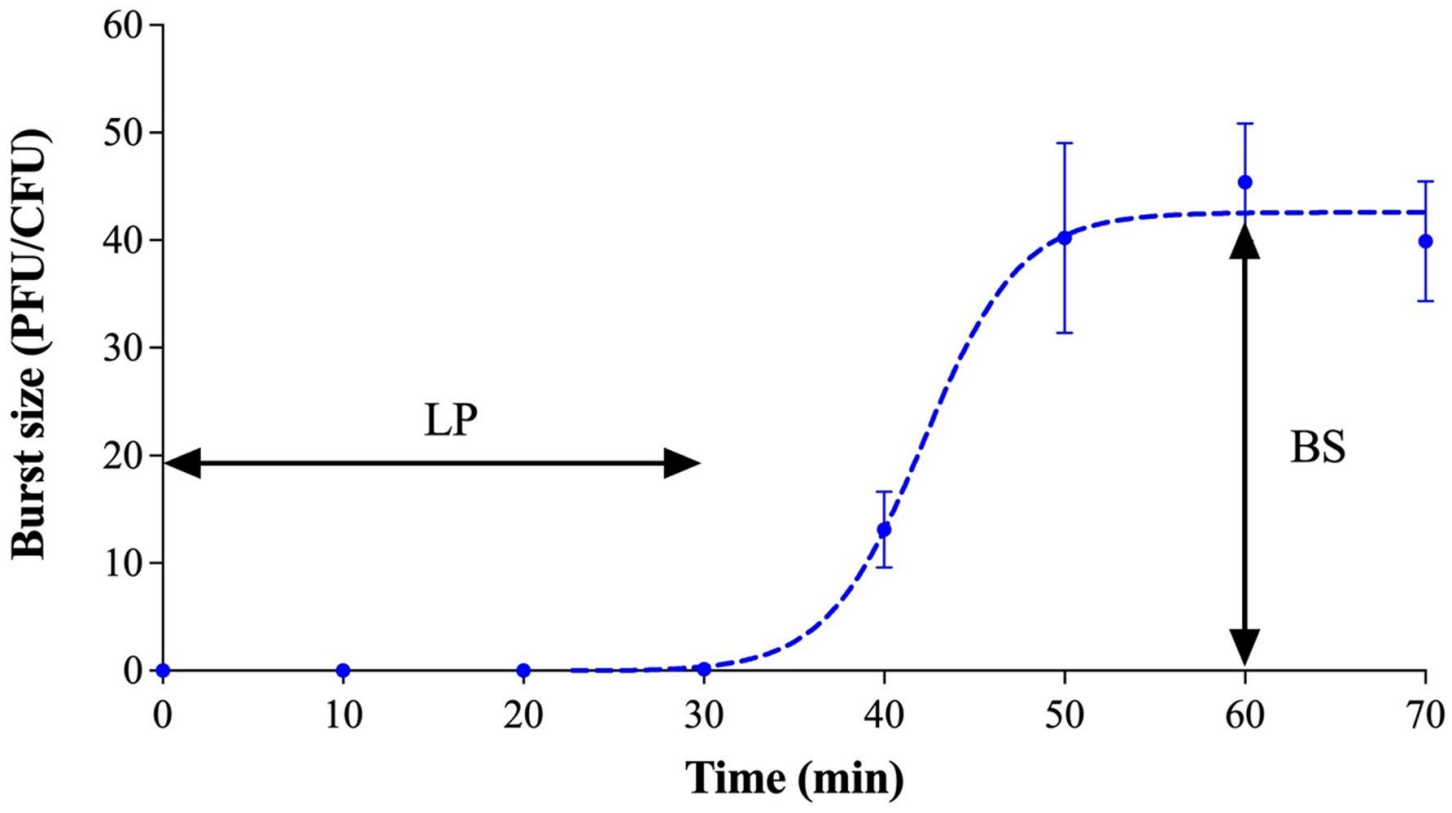
One-step growth curve of phage SIY1lw infecting *S.* Infantis (RM2481). The growth parameters of the phage indicate a latent period (LP) of 30 min and an average burst size (BS) of 42 phages per infected cell. The error bars present the standard error of the mean (SEM) for each time point of the one-step growth curve.

Phage vB_SalS-SIY1lw (or SIY1lw) has a siphovirus morphology that contains an icosahedral head of 76 ± 5 nm in diameter and a long non-contractile tail of 178 ± 7 nm in length (Fig. 1). A central tail fiber protein, similar to T5-like phage DT57C, was observed at the tip of the tail structure^16^. The growth factor of phage SIY1lw on *S.* Infantis (RM2481) showed that the phage had a latent period of 30 min with an estimated burst size of 42 PFU/CFU (Fig. 2).

### Temperature, pH, and storage stability

**Fig. 3:**
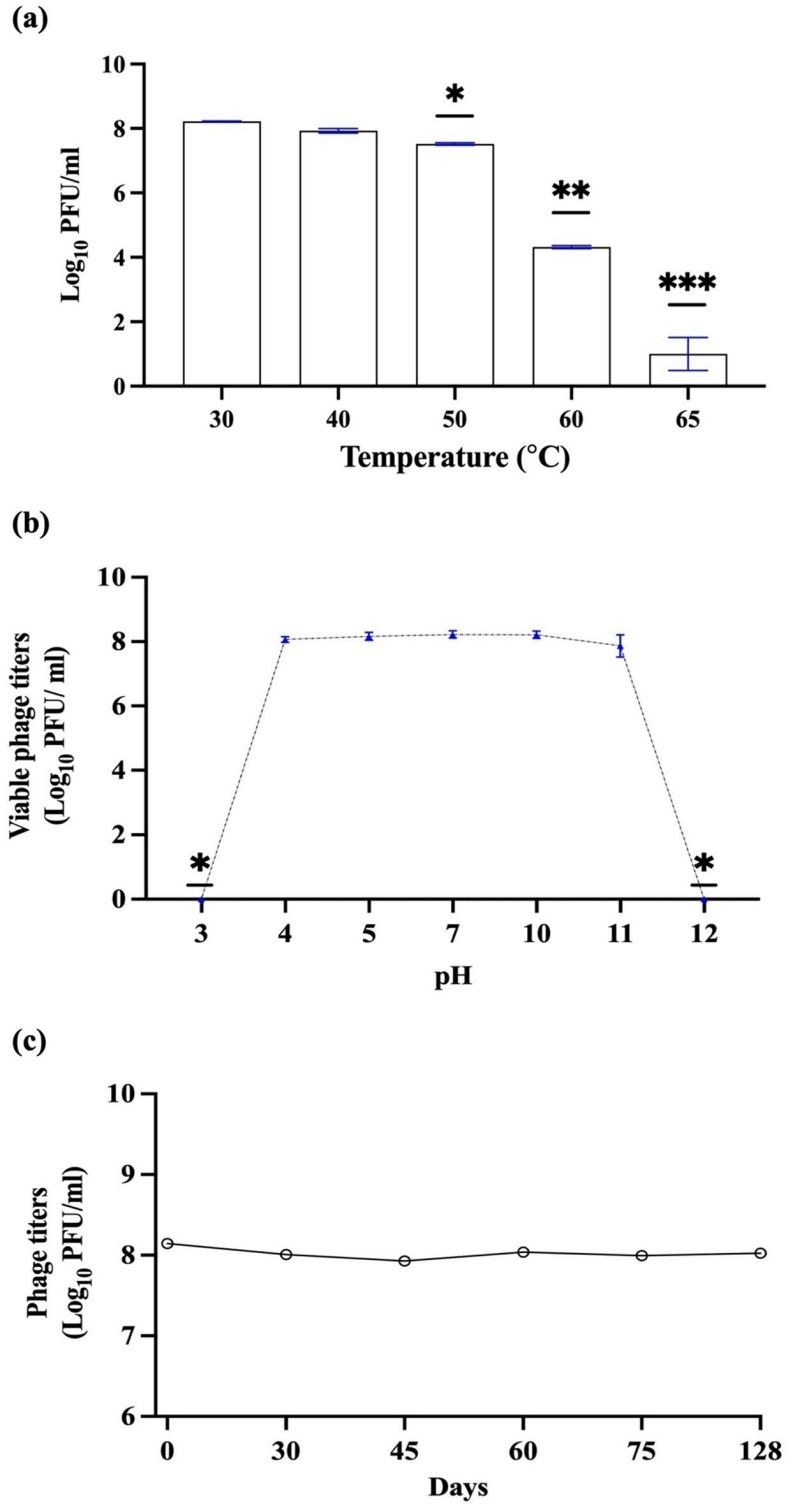
Stability of phage SIY1lw at (**a**) different temperatures (30 °C to 65 °C) for 24 h, (**b**) various pH (pH 3, pH 5, pH 7.5, pH 9, pH 10.5, and pH 12) at 30 °C for 24 h, and (**c**) 4 °C storage for 128 days. No statistical differences were observed between each time point of 4 °C storage. The means of phage titers that contain different numbers of asterisks differ (*P* < 0.05). The error bars show the SEM.

For the thermal stability test, SIY1lw was stable up to 40 °C but slightly decreased at 50 °C (*P* < 0.05). The phage titers further dropped as temperature increased to 65 °C by over 7 log (Fig. 3a). Regarding pH tolerance, phage SIY1lw maintained similar virion levels from pH 4 to pH 11 for 24 h at 30 °C (Fig. 3b); however, the phage titer decreased below the detection levels at both pH 3 and pH 12. Additionally, the phage was very stable at refrigeration storage (4 °C) for 128 days without a significant drop in phage titers (Fig. 3c).

### Host range and productive infection of SIY1lw

Phage SIY1lw showed lytic infection against two *Salmonella enterica* serovars, including Infantis and Newport, and generic *Escherichia coli* strains (Table 1). The phage showed medium to high production efficiency among most *S.* Infantis and *S.* Newport strains but low production efficiency against *S.* Infantis RM2480, FSIS9799, FSIS4897, and FSIS7821. *S.* Infantis FSIS9851 and RM19091 were resistant to the phage infection. Furthermore, SIY1lw demonstrated high productive infection against generic *E. coli* ATCC 13706 and ATCC 15597 but did not infect any STEC, *E. albertii* strains, and TVS353, which was non-pathogenic environmental isolate (Table 1).

**Table 1:** Host range and EOP of phage SIY1lw against various pathogenic *E. coli* and *Salmonella* strains. *EOP was calculated by the ratio of phage titer on a test bacterium versus the primary bacterial host. High production efficiency is EOP ≥ 0.5, medium production efficiency is 0.5 > EOP ≥ 0.1, low production efficiency is 0.1 > EOP > 0.001, and the inefficiency of phage production is EOP ≤ 0.001. H means the bacterial host used for the phage isolation and propagation. R means the bacterial strain resistant to the phage infection without any bacterial lysis.

Strains	Strain Ref. No.	EOP
*Salmonella*	*Salmonella* Infantis (BAA-1675)	1.29
	*Salmonella* Infantis (RM2480)	0.08
	*Salmonella* Infantis (RM2481)	H
	*Salmonella* Infantis (RM19091)	R
	*Salmonella* Infantis (RM19096)	0.15
	*Salmonella* Infantis (FSIS9799)	0.09
	*Salmonella* Infantis (FSIS9916)	0.15
	*Salmonella* Infantis (FSIS4897)	0.05
	*Salmonella* Infantis (FSIS4900)	0.11
	*Salmonella* Infantis (FSIS4921)	0.31
	*Salmonella* Infantis (FSIS9851)	R
	*Salmonella* Infantis (FSIS9861)	0.26
	*Salmonella* Infantis (FSIS7821)	0.05
	*Salmonella* Infantis (FSIS5221)	R
	*Salmonella* Infantis (FSIS7823)	0.31
Other *S.* serovars	*Salmonella* Typhimurium 14028	R
	*Salmonella* Montevideo S1	R
	*Salmonella* Newport H1073	0.34
	*Salmonella* Heidelberg 45955	R
	*Salmonella* Enteritidis PT-30	R
STEC	O26, O45, O103, O111, O121, O145 & O157	R
*E. albertii*	*E. albertii* RM9973, RM9974, & RM15113	R
Non-pathogenic *E. coli*	ATCC 13706	0.77
	ATCC 15597	0.6
	TVS 353	R

### Genomic analysis

**Fig. 4:**
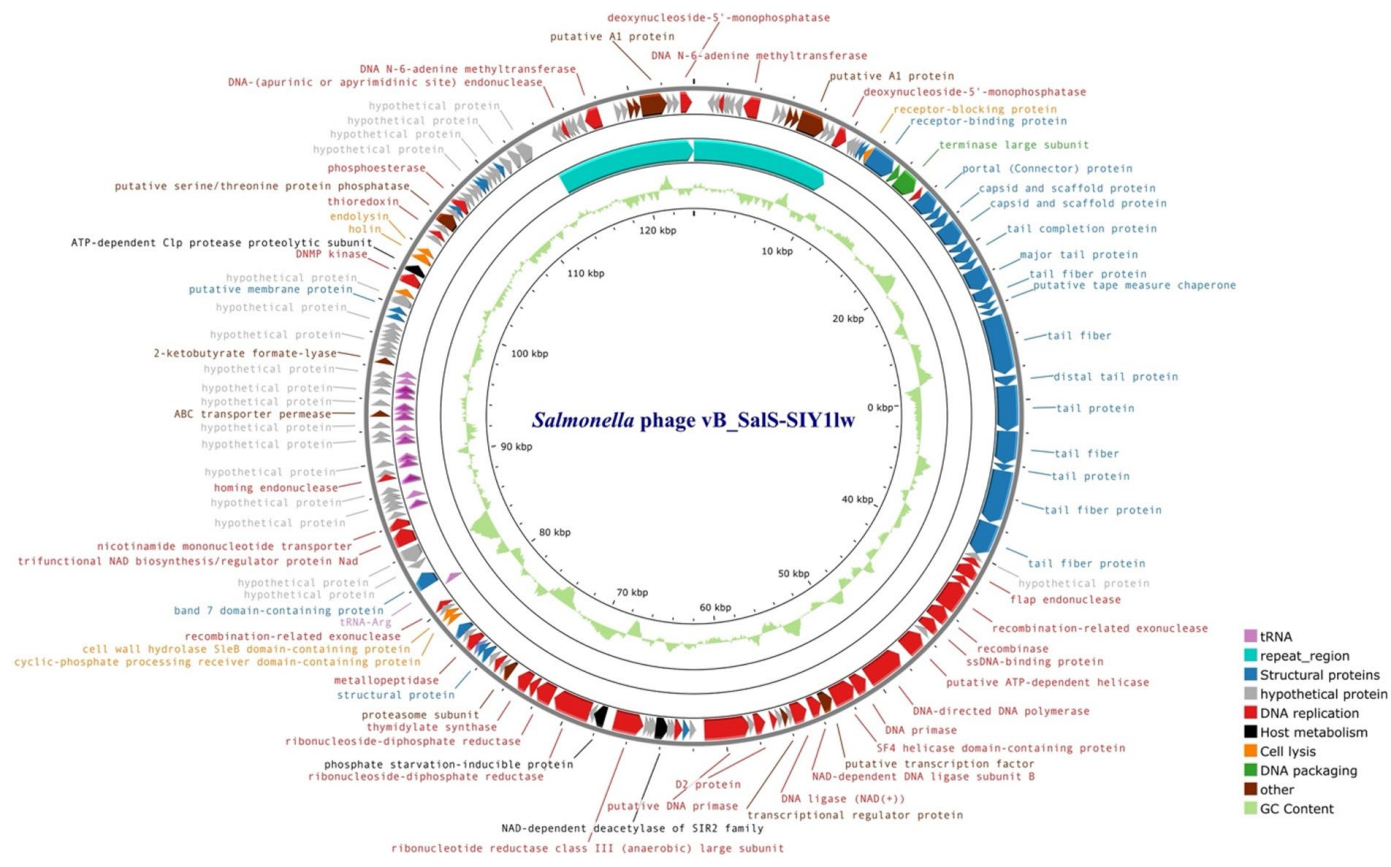
The genome map of *Salmonella* phage vB_Sal-SIY1lw (or SIY1lw) generated using the CGview server beta. The annotated ORFs are colored purple (tRNA), turquoise (repeat region), blue (structure proteins), grey (hypothetical protein), red (DNA replication), black (host metabolism), orange (cell lysis), dark green (DNA packaging), and brown (other), and those with known functions are labeled on the map. The center of the genome map provides % GC content (light green).

**Fig. 5:**
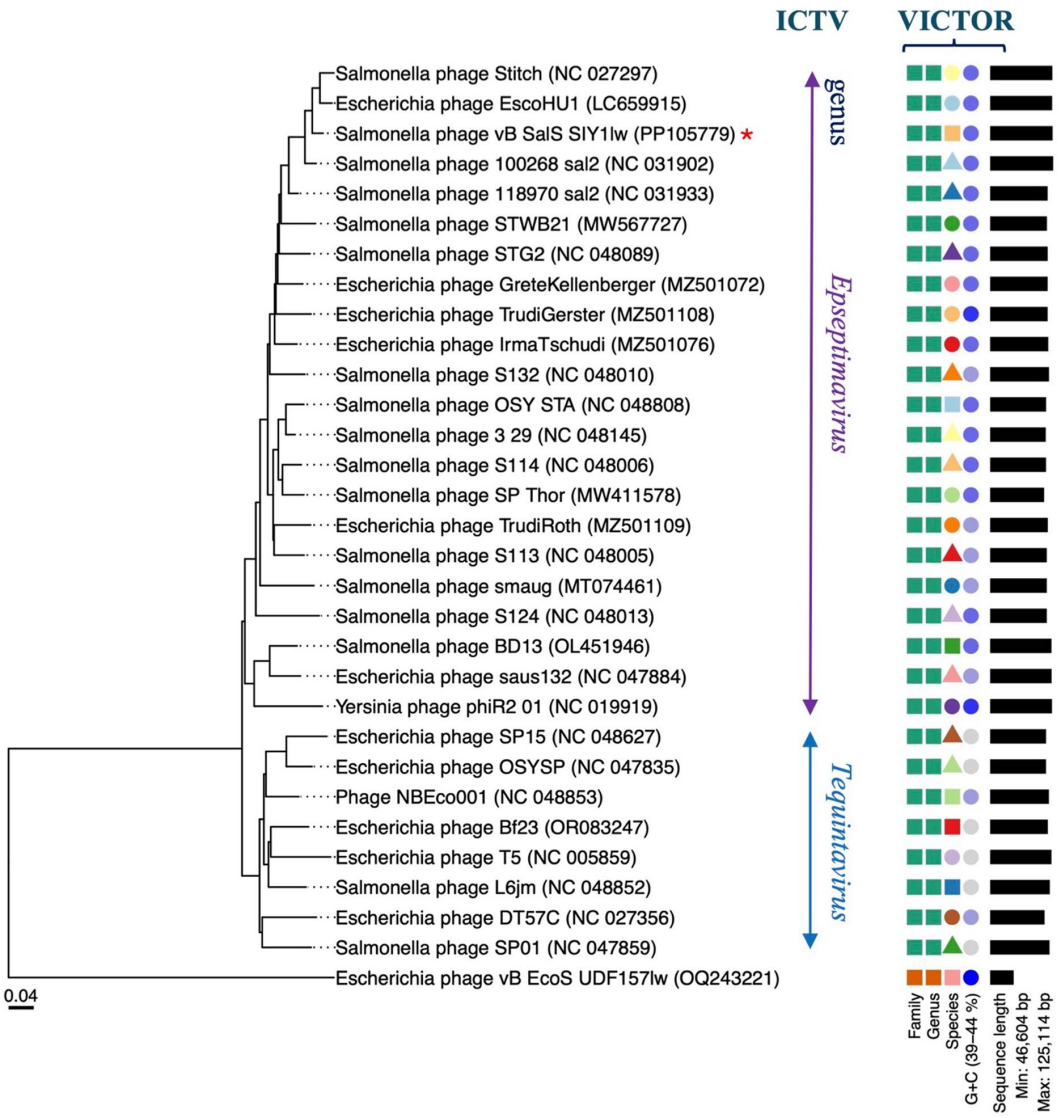
Whole genome sequence-based phylogenetic tree constructed using VICTOR (formula d0) on SIY1lw (with a red star) and the reference phages belonging to the *Epseptimavirus* and *Tequintavirus* genera, both under the *Demerecviridae* family. *Escherichia* phage vB_EcoS-UDF157lw used as an outgroup. The family, genus, and species are classified into different clusters with colors based on VICTOR analysis. Genomic GC content and sequence length are represented in the hue of color and horizontal black lines, respectively, on the right side of the tree.

SIY1lw contained a double-stranded DNA phage with a genome size of 123,932 bp and an average GC content of 40.3% and encoded 198 open reading frames (ORFs), of which 92 were annotated with known functions and 27 tRNAs, with long terminal repeats of ~ 10.1 kbp (Fig. 4). The predicted functions of known ORFs included phage structural proteins (capsid and tail proteins), bacterial host binding proteins (receptor-binding and tail fiber proteins), host lysis (holin, endolysin, and spanin), phage DNA replication and packaging, host cell regulation and metabolism, and other functions (Table S1). The phylogenetic analysis based on whole-genome sequences via the Virus Classification and Tree Building Online Resource (VICTOR) analysis demonstrated phage SIY1lw was in its own clade at the nucleic acid level with 22 reference phages belonging to the *Epseptimavirus* genus under the *Demerecviridae* family (Fig. 5). In addition, the pairwise comparison results indicated that SIY1lw shared an average nucleotide identity calculated based on BLAST+ (ANIb) of 95.75%, 95.96%, 96.18%, and 96.23% to reference *Epseptimavirus* phages of *Escherichia* phage EscoHU1, *Salmonella* phage OSY-STA, *Salmonella* phage Stitch, and *Salmonella* phage 100268_sal2, respectively. As a result, phage SIY1lw belonged to the *Epseptimavirus* genus due to more than 70% nucleotide identity coverage over the full genome length of the reference phages^17^. In silico analysis showed that SIY1lw contained no ORFs associated with lysogeny, bacterial virulence, and antibiotic resistance. DeepPL analysis showed that SIY1lw had a lytic lifecycle.

**Fig. 6:**
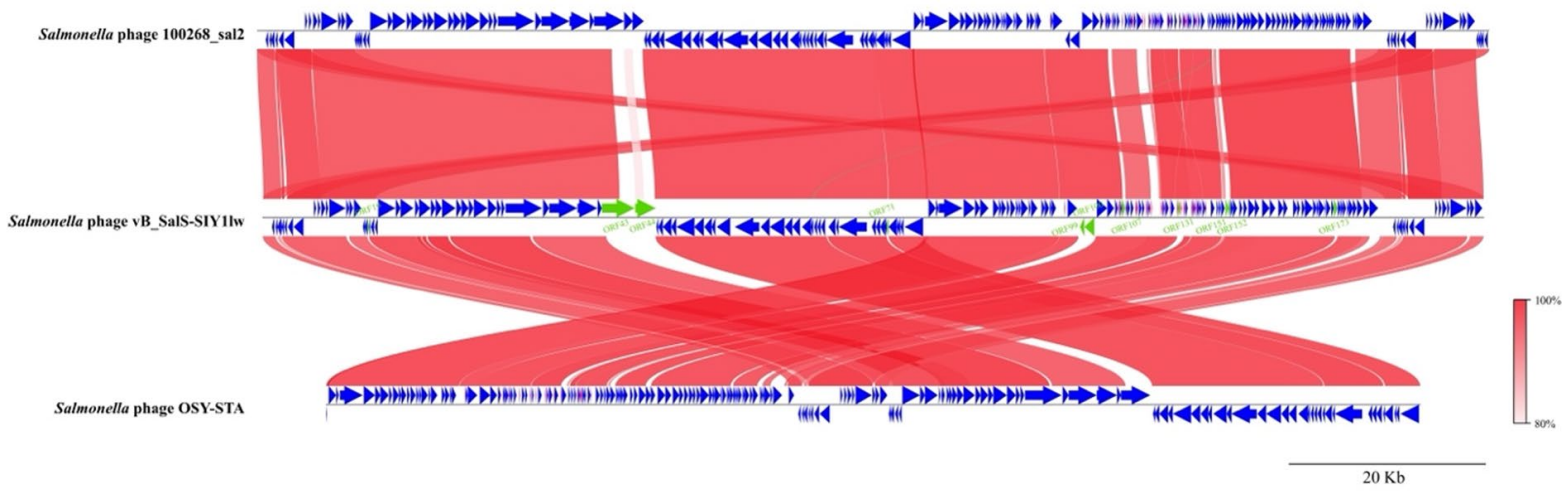
Whole-genome pairwise comparison of SIY1lw and its close-related *Salmonella* phages 100268_sal2 and OSY-STA using pyGenomeViz. The whole genome map is illustrated as blue arrows with the order of annotated ORFs from left to right along the phage genome. Regions of sequence similarity are connected by a red-scale shaded area. Eleven ORFs with green highlights from SIY1lw are absent on either phage 100268_sal2 or OSY-STA genome. The sequence direction of phage 100268_sal2 is adjusted before the analysis. ORF 43, ORF 44, and ORF 131 encode tail fiber, tail fiber, and ABC transporter permease, respectively; the other ORFs encode hypothetical proteins.

**Fig. 7:**
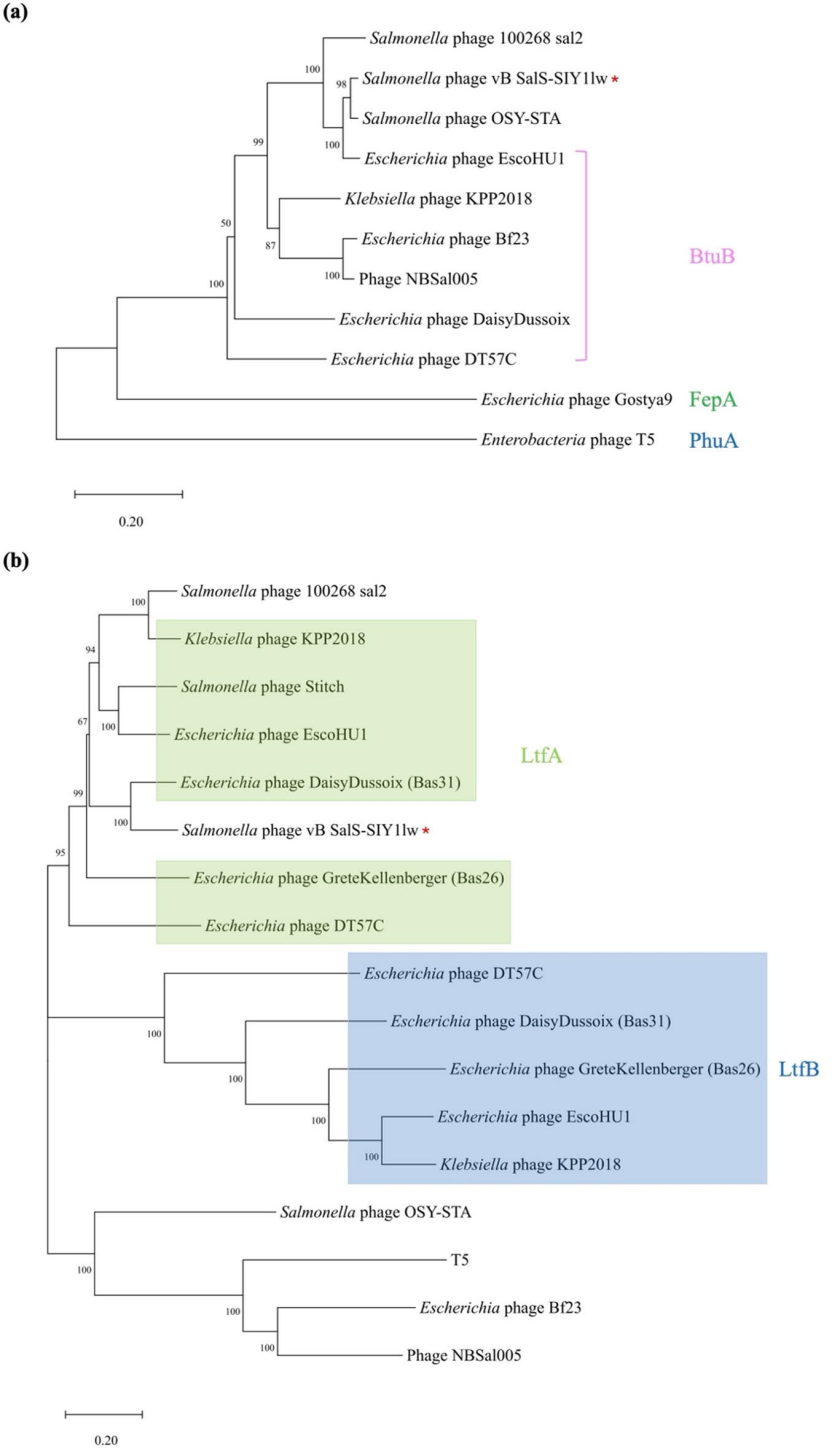
Phylogenetic analysis of the nucleotide sequences encoding the (**a**) receptor-binding protein (ORF 23) and (**b**) tail fiber protein (ORF 43) for phage SIY1lw and its close-related reference phages. Membrane transport proteins, FepA, PhuA, and BtuB, serve as primary receptors of the reference phages. Phage SIY1lw is indicated with a red asterisk.

Based on the whole-genome alignment, the results showed that SIY1lw contained 5 ORFs—ORF 43, ORF 44, ORF 107, ORF 151, and ORF 152— and 8 ORFs, including ORF 19, ORF 43, ORF 44, ORF71, ORF 99, ORF 100, ORF 131, and ORF 173, that were absent on the phages 100268_sal2 and OSY-STA, respectively (Fig. 6). Although most ORFs did not have known function, the primary difference fell on ORF 43 and ORF 44, both encoding tail fiber proteins related to the phage host range. The phylogenetic analysis of the receptor-binding protein (RBP) showed that phage SIY1lw shared high nucleotide sequence similarity with that in *Salmonella* phage OSY-STA (Fig. 7a). According to the CoreGene analysis, the tail fiber, encoded by ORF 43, in SIY1lw was similar to the L-shape tail fiber (Ltf), which was commonly found among T5-like phages closely associated with host recognition and receptor binding. The phylogenetic results demonstrated that SIY1lw was in a close evolutionary relationship at the nucleotide sequence with the LtfA in *Escherichia* phage Daisy Dussoix (Bas31) (Fig. 7b). Additionally, among the phages containing single Ltf protein (without both LtfA and LtfB), SIY1lw had a distinct nucleotide sequence similarity of Ltf-like protein from that of phages OSY-STA, T5, Bf23, and NBSal005 (Fig. 7b). Although *Salmonella* phage 100268_sal2 shared a high nucleotide sequence similarity with *Klebsiella* phage KPP2018, its Ltf classification was still unclear.

### Phage protein analysis

**Fig. 8:**
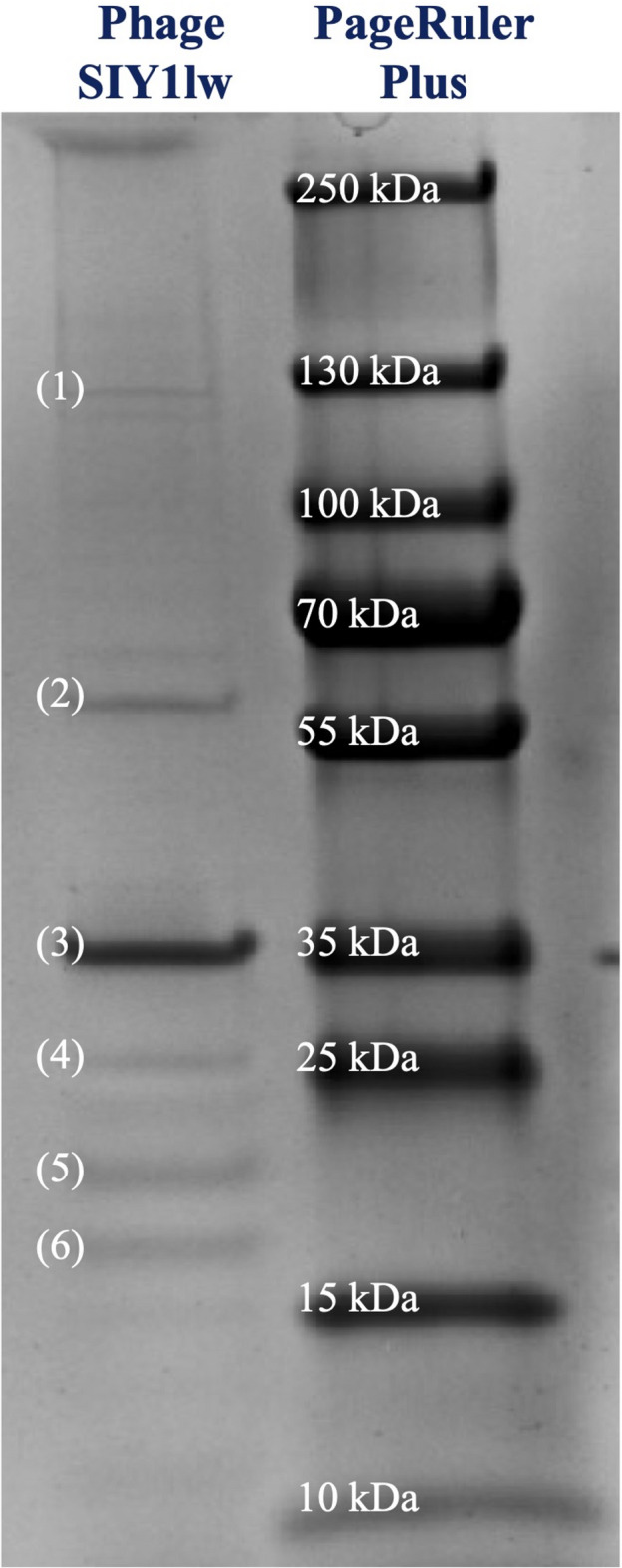
The phage SIY1lw proteins on a 12% SDS-PAGE gel. (1) = tail protein; (2) = major tail protein; (3) = capsid and scaffold protein; (4) = putative transcription factor and tail fiber protein; (5) = head completion protein; (6) = neck whiskers protein. Refer to Table 2 for the corresponding ORFs and the rest of the protein information.

Six bands related to phage SIY1lw proteins were separated through sodium dodecyl sulfate-polyacrylamide gel (SDS-PAGE), with molecular weights ranging from approximately 17 to 107 kDa (Fig. 8). The identified gel bands, labeled with 1 to 6, included multiple structural phage proteins, such as tail protein, tail fiber protein, capsid and scaffold protein, major tail protein, putative transcription factor, head completion protein, and neck whiskers protein. On the other hand, the protein of putative transcription factor was also found in band 4, the same as the tail fiber protein (ORF 92). The calculated mass of the capsid and scaffold protein was about 50.59 kDa; however, the actual protein was identified in band 3 with an approximate size of 35 kDa. This finding could be due to the cleavage of the scaffold portion of the protein by a maturation protease^18^. The coverage of amino acid sequences for these detected proteins ranged from 15 to 51% by mass spectrometry (Table 2). All identified protein bands matched the annotation results of the phage SIY1lw genome (Table 2).

**Table 2:** Phage protein analysis of SIY1lw, identified by high-performance liquid chromatography with tandem mass spectrometry (HPLC-MS-MS). Calculated monoisotopic masses were obtained from the predicted amino acid sequences, not the mature proteins.

Gel band	ORF	Predicted function	Calculated monoisotopic mass (MW kDa)	No. of peptides	Sequence coverage
1	SIY1lw_00040	Tail protein	107.42	15	20%
2	SIY1lw_00034	Major tail protein	50.35	5	15%
3	SIY1lw_00030	Capsid and scaffold protein	50.59	8	20%
4	SIY1lw_00057	Putative transcription factor	27.02	9	49%
4	SIY1lw_00092	Tail fiber protein	23.96	4	23%
5	SIY1lw_00031	Head completion protein	19.46	4	37%
6	SIY1lw_00028	Neck whiskers protein	17.04	6	51%

### Antimicrobial activity

**Fig. 9:**
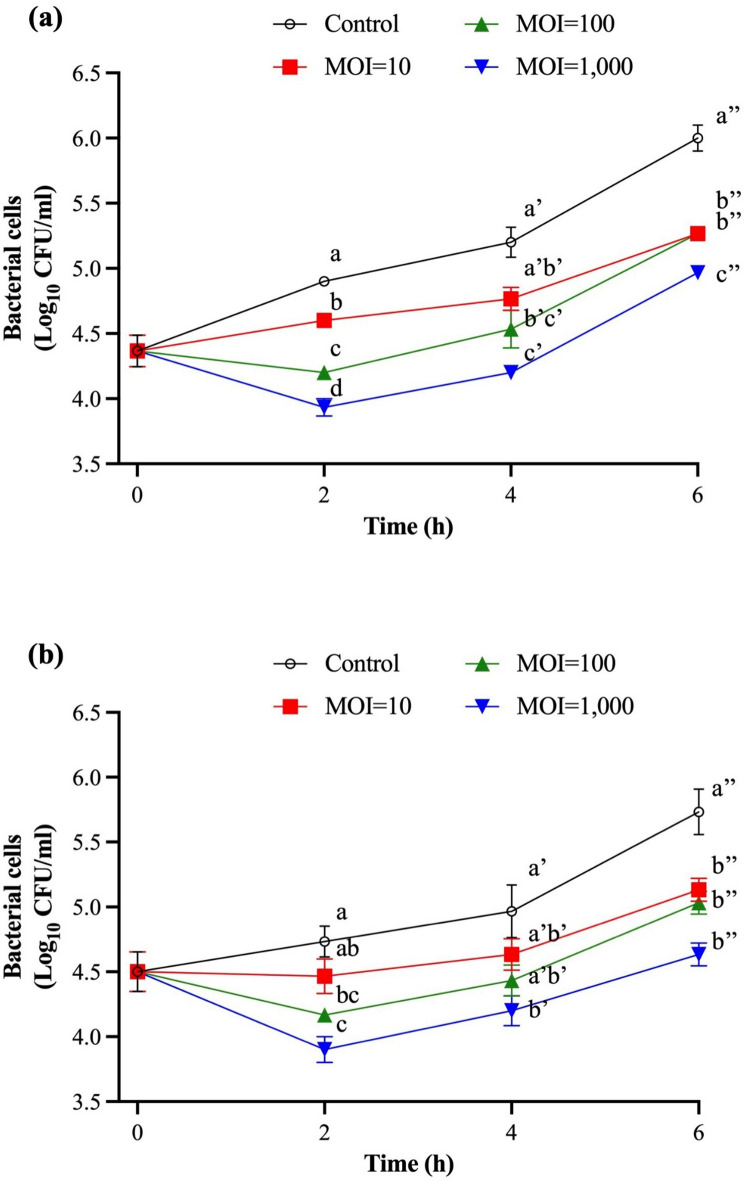
The antimicrobial activity of SIY1lw with various MOIs (10, 100, and 1000) against (**a**) *S.* Infantis (FSIS7823) and (**b**) *S.* Infantis (FSIS4921) in LB at 25°C for 6 hours. The control group only contains bacterial culture. Means of phage titers within each time point lacking common letters (a, b, c, and d; a′, b′, and c′; or a″, b″, and c″) differ (*P* < 0.05). The error bars show the standard error of the mean (SEM).

The in vitro antimicrobial activity of SIY1lw against multidrug-resistant *S.* Infantis (FSIS7823 and FSIS4921) strains was determined in lysogeny broth at 25 °C. The results showed that two *S.* Infantis strains treated with MOI of 10 were mostly inhibited at the levels without significant difference from the control group within the first 4 h of the treatment (Fig. 9). Regarding MOIs of 100 and 1,000, the bacterial populations were reduced to the lowest levels, with 0.7 and 1.0 log reduction for *S.* Infantis FSIS7823 and 0.6 and 0.8 log reduction, respectively, for *S.* Infantis FSIS4921 compared to the control group, in 2 h and started to bounce back afterward until the end of the treatment period. Additionally, the maximum bacterial reductions of *S.* Infantis (FSIS7823) were observed at the 2-h and 4-h time points by 1 log compared to the control group (*P* < 0.05) (Fig. 9a); *S.* Infantis (4921) was reduced by 0.8 log at the 2-h and 4-h time points and reached the maximum reduction of 1.1 log within 6-h of the treatment (*P* < 0.05) (Fig. 9b). These results demonstrated that the antimicrobial effects were proportional to the high MOI used, with the MOI of 1,000 being the most effective dosage.

## Discussion

*Salmonella enterica* strains have caused numerous foodborne outbreaks and are highly associated with antibiotic resistance, posing a greater risk to the public. Some serovars, such as *S.* Infantis, are antibiotic-resistant and can tolerate external stresses, such as heat, osmotic pressure, or acid, deriving from many disinfection methods^19^, as planktonic cells or with enhanced biofilm-forming ability^20^. These factors enable the pathogens to reduce the antimicrobial impact from chemical-based interventions commonly used in the food industry, resulting in the spread of contamination. The U.S. Food and Drug Administration (FDA) has approved several phage products as Generally Recognized As Safe (GRAS), promoting safety awareness and phage applications for improving food safety^21,22^.

This study characterized a newly isolated phage SIY1lw via genomic and biological approaches for the antimicrobial potential of multidrug-resistant *S.* Infantis strains. The biological features show that phage SIY1lw has a burst size of 42 PFU/CFU and a shorter latent period than other *S.* Infantis-infecting phages against *S.* Infantis strain^23^. Despite having a small burst size, phage SIY1lw with a relatively short latent period may quickly carry out multiple parallel infections against the target bacteria if the bacterial concentration is high^24^. Phage SIY1lw revealed a high tolerance within a wide range of pH and refrigerated and thermal temperatures, and these biological stability features enable SIY1lw to be sustainable under various food-associated conditions. Regarding the host range, phage SIY1 demonstrates higher EOPs against the same panel of *S.* Infantis strains than phage a *Tequintavirus* phage SIA3lw, previously isolated by our lab^25^. The phenomenon could be due to the lack of robust anti-restriction activity in *Epseptimavirus* phages instead of its sister genus *Tequintavirus*, making SIY1lw susceptible to host restriction-modification systems^26,27^.

The phylogenetic results demonstrate that SIY1lw is a new member of the phages belonging to the *Epseptimavirus* genus. Phage SIY1lw contained a divergent region with two tail fiber proteins (ORF 43 and ORF 44) not found in the reference *Salmonella* phages 100268_sal2 and OSY-STA, both targeting *S.* Typhimurium strains. Phage SIY1lw RBP clustered with *Salmonella* phage OSY-STA, but was distinct from *Escherichia* phage EscoHU1, which used BtuB as the receptor. However, the outer membrane receptors used by SIY1lw are unclear because those used by *Salmonella* phage OSY-STA are not yet known. The phylogenetic analysis revealed that phage SIY1lw had an LtfA-like protein, which could recognize a similar O-antigen as *Escherichia* phage DaisyDussoix did^28^. On the contrary, SIY1lw did not carry a LtfB protein to target different parts of lipopolysaccharide (LPS), possibly resulting in a different host range against *E. coli* strains from *Escherichia* phage Daisy Dussoix (Bas31) containing both LtfA and LtfB. Furthermore, SIYlw contained the gene encoding a band 7 domain-containing protein, which might have a similar function as lipoprotein found in most T5-like phages^29^; the primary function of the protein is to bind to FhuA and prevent the released phage progenies from binding to lysed cell if the phages use FhuA as the primary receptor. Nevertheless, the host receptors and the function of Ltf-like protein for phage SIY1lw require further studies to confirm its polyvalent effects against non-pathogenic *E. coli* and *Salmonella enterica* (serovars of Infantis and Newport). Most importantly, SIY1lw did not carry virulence, lysogenic, or antibiotic-resistant genes, thus making it an ideal candidate for use as a potential antimicrobial agent.

For antimicrobial testing, SIY1lw with low MOI did not yield sufficient in vitro antimicrobial effects against *S.* Infantis until higher MOIs were used in this study. The phenomenon is likely due to the small burst size of SIY1lw, which may contribute to the equivalent rates of phage infection and target bacterial growth. Thus, higher initial phage titers are much more critical in bacterial reduction than those from the subsequent infection by the phage progenies produced from a previous infection cycle. On the other hand, super-infection can lead to lysis inhibition by phages^30^ and may vary among different *Salmonella* serovars; the phenomenon could be one of the factors contributing to no bacterial reduction of *S.* Newport different from that of both *S.* Infantis strains after the treatment with SIY1lw at an MOI of 10 in this study (Fig. S1). Moreover, the bacterial regrowth of the phage-treated *S.* Infantis after 2 h of the treatment at similar MOIs (100 and 1,000) is likely due to the development of bacteriophage-insensitive mutants^25^, in which a mutation may not necessarily evolve to genetic level^31^. However, developing a phage cocktail maybe a solution to overcome phage resistance^32^. To reduce the contamination of *S.* Infantis from poultry, a future study is required to test the efficacy of phage SIY1lw in lowering the bacterial level in the broiler’s gastrointestinal (GI) tract^33^. Additional protection may be necessary to increase the phage viability to sustain the low pH environment of the broiler’s GI tract via oral delivery^34^.

To conclude, *Salmonella* phage vB_SalS-SIY1lw is a new *Epseptimavirus* phage showing polyvalent effects against *Salmonella enterica* and *E. coli* strains. Most importantly, the phage can reduce multidrug-resistant *S.* Infantis within 2 h of treatment. These findings shed light on phage SIY1lw as an alternative antimicrobial agent for antibiotic resistance issues in the food industry. Additionally, biological features allow SIY1lw to be used for hurdle intervention or further application development to improve the overall antimicrobial efficacy.

## Methods

### Bacteriophage isolation

*Salmonella* phage SIY1lw was isolated and purified from sewage water collected near Davis, California, using the bacterial host of *S.* Infantis RM2481 following the methods previously described^25^. The phage was propagated with an overnight culture of *S.* Infantis RM2481 strain in 40 mL tryptic soy broth (TSB; Difco, Becton Dickinson, Sparks, MD, USA) containing 10 mM CaCl_2_ at 37 °C for 24 h. Subsequently, centrifugation at 8,000 ×*g* for 5 min and 0.22-µm filtration was used to remove bacterial debris of the phage lysate. The propagated phage was subjected to concentration via a 50 kDa cut-off Amicon Ultra-15 Centrifugal Filter Unit (Merck Millipore, Billerica, MA, USA) and purification through CsCl gradient ultracentrifugation to remove bacterial debris and DNA based on the methods previously described^35^. The purified phage was used for morphology observation under microscopy and DNA extraction for whole genome sequencing.

### Bacterial strains

Generic *E. coli* (ATCC 13706 and ATCC 15597), environmental isolate of TVS353, pathogenic Shiga toxin-producing *E. coli* (STEC, the serogroups of O26, O45, O103, O111, O121, O145, and O157), *E. albertii*, and *Salmonella enterica* (Typhimurium, Enteritidis, Montevideo, Newport, Heidelberg, and Infantis) strains were obtained from the Produce Safety and Microbiology (PSM) Research Unit (Table S2) at the U.S. Department of Agriculture (USDA), Agricultural Research Service (ARS), Western Regional Research Center, Albany, CA, USA. These strains were used to determine the host range and efficiency of plating for the phage isolated in the current study. *S.* Infantis (RM2481), the primary host of phage isolation, was used for phage propagation, quantification, and the one-step growth curve study. Each bacterial strain was prepared by inoculating 10 mL of TSB with a loopful of bacterial strain for 20-h incubation at 37 °C before use.

### One-step growth curve

*S.* Infantis (RM2481) was used to determine the growth factor of phage SIY1lw using the previous method^36^. A log-phase bacterial solution was prepared after the subculture of an overnight culture in 20 mL TSB for 2-h incubation at 37 °C. Subsequently, the phage was mixed with the bacterial solution at an MOI of 0.01 and supplemented with CaCl_2_ at 10 mM for 10-minute incubation at 37 °C, ensuring phage adsorption onto the bacterial membranes. The mixture was centrifuged at 10,000 ×g for 5 min at 4 °C before discarding the supernatant. The bacterial pellet was washed with 2 mL TSB twice and resuspended in 20 mL sterile TSB. An aliquot of 0.3 mL resuspension was added to 29.7 mL TSB and incubated at 37 °C to start the clock for the experiment. Meanwhile, the phage-infected cells of *S.* Infantis were determined right at time 0 of the incubation period by mixing 50 µL of the final 30-mL phage-infected bacterial solution (no filtration) with 100 µL of fresh overnight bacterial culture using a double-layer plaque assay^37^. Later, the phage-infected bacterial solution was subjected to a 1 mL sampling every 5 min for filtration through a 0.22-µm filter membrane. The titers of phage SIY1lw at each time point were determined using the double-layer plaque assay described above. The experiment was conducted in three replications to estimate the latent period—the time required from phage infection to bacterial cell lysis to detect phage progenies—and the burst size of phage SIY1lw.

### Phage stability (pH, temperature, and storage)

The pH test was conducted on phage SIY1lw using the method previously described^38^. In brief, phage SIY1lw (~ 10 log PFU/mL) with 100 µL was added into 4.9 mL of SM buffer (Thermo Fisher Scientific, Waltham, MA, USA) to reach the final pH values of 3, 4, 5, 7, 10, 11, and 12. Samples were incubated at 30 °C for 24 h and subsequently subjected to viable phage enumeration using the double-layer plaque assay. Regarding the thermal stability test, SIY1lw solution was prepared in SM buffer to reach approximately 8 log PFU/mL and dispensed with 0.5 mL of the phage solution in several sterile microcentrifuge tubes. The phage was placed in different water baths set to 30 °C (control), 40 °C, 50 °C, 60 °C, and 65 °C for 24-h thermal treatment. The temperatures used in this study covered possible environmental conditions in either pre-harvest or post-harvest food processing settings. The remaining phage concentrations were then determined using the double-layer plaque assay. For the storage test, phage SIY1lw with 8 log PFU/mL was prepared in SM buffer before aliquoting 0.5 mL phage per tube in multiple sterile microcentrifuge tubes and then stored at 4 °C for 128 days. All experiments were conducted in three replications.

### Host range and efficiency of plating

The host range test of SIY1lw against non-pathogenic *E. coli*, seven primary STEC serogroups, *E. albertii*, and various *Salmonella enterica* serovars, primarily Infantis, strains was determined using a spot test assay as previously described^38^. The tested bacterial strains that showed lysis were further subjected to the efficiency of plating (EOP) assay to determine productive infection of phage SIY1lw by measuring the phage progenies produced from the tested strains versus those produced from the host strain of *S.* Infantis (RM2481)^39^. Briefly, a fresh culture of the selected bacteria was prepared in TSB at 37 °C overnight for phage SIY1lw enumeration with four successive dilutions of the phage (10^− 3^ to 10^− 7^) using the double-layer plaque assay. The plates were incubated at 37 °C for 20 h for plaque count and EOP calculation. The experiment was conducted in three replications. Generally, a high phage-producing efficiency had an EOP of 0.5 or more; a medium-producing efficiency had an EOP above 0.1 but below 0.5; a low-producing efficiency had an EOP between 0.001 and 0.1; inefficient phage production was any value lower than 0.001.

### Transmission electron microscopy

The morphology of phage SIY1lw was determined under the transmission electron microscope FEI Tecnai 12 120 kV TEM (FEI, Hillsboro, OR, USA) using the method previously described^36^. In brief, an aliquot of 5 µL CsCl-purified phage was mixed with 5 µL sterile water for the negative staining preparation prior to observation.

### Genomic analysis

The CsCl-purified SIY1lw was used for phage DNA extraction via a Norgen phage DNA extraction kit (Thorold, ON, Canada). DNA library preparation was prepared based on the previously published protocol^40^ and subsequently loaded on a MiSeq Reagent Kit v2 (500-cycle) for whole-genome sequencing using the MiSeq platform (Illumina, San Deigo, CA, USA). Raw sequence reads of 13.3 million 2 × 250-bp paired-end reads were subjected to Trimmomatic v.0.36 with the setting of Q30 to remove poor-quality reads. The SPAdes^41^ (Galaxy Version 4.2.0), with the default settings, was used for de novo assembly of the remaining quality reads. The resulting contig with N_50_ contig length of 113,831 bp (also the largest contig) was confirmed as a phage genome via BLASTn before being subjected to the PhageTerm analysis (Galaxy v.1.0.12) to predict the packaging mechanisms and termini of the phage genome^42^. Later, the re-organized phage sequence was annotated through Prokka pipeline Galaxy 1.13^43^, with the default settings; the annotation was confirmed with the Universal Protein Resource (UniProt) database^44^ and curated using Geneious Primes (version 2024.0.3). tRNAscan-SE (v2.0) was used to predict tRNAs in the phage genome^45^. Moreover, the VirulenceFinder v2.0^46^ (https://cge.food.dtu.dk/services/VirulenceFinder/; accessed on 1/23/2025) and ResFinder v4.6.0 Webservers^47^ (https://cge.food.dtu.dk/services/ResFinder/; accessed on 11/19/2024) were used for virulence and antibiotic resistance genes, respectively, with the default settings. DeepPL was used to predict the phage lifecycle of SIY1lw^48^.

The Virus Classification and Tree Building Online Resource (VICTOR) webserver (https://ggdc.dsmz.de/victor.php) was used for the whole-genome sequence phylogenetic analysis of SIY1lw and 30 reference phages belonging to the *Epseptimavirus* and *Tequintavirus* genera, both under the *Demerecviridae* family^49^. The selected phages from the *Epseptimavirus* and *Tequintavirus* genera were confirmed by the International Committee on Taxonomy of Viruses (ICTV); the siphovirus UDF157lw was used as an outgroup of the phylogenetic tree. The genome comparison between SIY1lw and its close reference phages was visualized using the pyGenomeViz v.0.5.1. Average nucleotide identity among phage SIY1lw and the selected reference phages was calculated based on BLAST+ (ANIb) using the JSpeciesWS web server^50^. The conservative (core) genes of SIY1lw compared to the close-related reference phages, such as *Salmonella* phages Stitch and 100268_sal2, were analyzed using CoreGenes3.5^51^. Phylogenetic analysis was conducted on the nucleotide sequences of ORF 23 and ORF 43 encoding a receptor-binding protein and tail fiber protein, respectively, as previously described^36^. Additionally, membrane transport proteins, such as FepA, PhuA, and BtuB, as primary receptors recognized by the receptor-binding proteins from the reference phages were obtained from other studies^26,52,53^. In brief, the nucleotide sequences were aligned using the Clustal Omega (v.1.2.3) on the Geneious Prime software (v.2024.0) installed on a local computer. The phylogenetic trees were performed using the MEGA11 program, with the maximum composite likelihood method and 500 bootstrap replications^54^.

### Phage protein analysis

The CsCl-purified phage SIY1lw lysate was subjected to gel electrophoresis, in-gel digestion with trypsin, followed by reverse phase nanoflow high-performance liquid chromatography with tandem mass spectrometry (HPLC-MS-MS), and data analyses as previously described^39^. First of all, the purified phage lysate was reduced with 0.5% 2-mercaptoethanol in Laemmli buffer per manufacturers’ instructions (Bio-Rad, Hercules, CA, USA) and subsequently subjected to SDS-PAGE using a 1D Bio-Rad 12% TGX gel (Bio-Rad, Hercules, CA, USA). The original SDS-PAGE image was illustrated in Fig. S2. After staining using ImperialTM Protein Stain (Thermo Fisher Scientific, Waltham, MA, USA), the gel lane was cut into several slices, each of which was placed into a reaction tray for in-gel digestion with trypsin (Promega, Madison, WI, USA) using a Digest Pro robot (Intavis, Köln, Germany). Sample digests were analyzed by nanoflow reversed-phase chromatography with an Eksigent NanoLC (Sciex, Framingham, MA, USA) using Picochip columns (New Objectives, Woburn, MA, USA). Data-dependent tandem mass spectra (MS-MS) were obtained in positive ion mode with an Orbitrap Elite mass spectrometer (Thermo Fisher Scientific, Waltham, MA, USA). MS-MS data were used to match the amino acid sequences derived from the whole-genome sequence of phage SIY1lw through Mascot software (Matrix Science, Boston, MA, USA). Protein identification parameters required a minimum of three identified peptides with a maximum mass error of 10 ppm for the parent ions.

### Antimicrobial activity against multidrug-resistant *S.* Infantis in lysogeny broth

*Salmonella* Infantis FSIS7823 and FSIS4921 strains, with different antibiotic resistance profiles (Table S3), were selected to evaluate the antimicrobial activity of SIY1lw following the method previously described with subtle modifications^37^. Briefly, bacterial culture was prepared in 10 ml TSB at 37 °C for 20 h and then diluted in lysogeny broth (LB; Invitrogen, Carlsbad, CA, USA) with approximately 4.4 log CFU/ml as final concentration to dispense 20-mL bacterial solution in sterile 50-mL conical tubes. At the same time, phage SIY1lw was diluted in SM buffer and then added to each tube to reach MOIs of 10, 100, and 1000, respectively. For the control group, an SM buffer with the same volume of the phage solution was added. All the tubes were incubated at 25 °C for 24 h, followed by bacterial plating on xylose lysine deoxycholate (XLD; Hardy Diagnostics, Santa Maria, CA, USA) overlayered thin tryptic soy agar (Thin Agar Layer Method, TAL)^55^ at the time points of 0, 2, 4, and 6 h to confirm bacterial concentration. The plates were incubated at 37 °C overnight for bacterial quantification. The experiment was conducted in three replications.

### Statistical analysis

Experiments were performed in three individual repetitions. Bacterial colony counts and phage titers were calculated as CFU/mL or PFU/mL and logarithmically transformed for statistical analysis. One-way analysis of variance (ANOVA) with statistical significance at a 5% level was used to evaluate the effects of stability tests (pH, temperature, and storage tests) on the phage enumeration. One-way ANOVA with Tukey HSD test (significance level of 5%) was used to determine in vitro antimicrobial activity among various MOIs on the bacterial reduction at each time point.

## Supplementary Information

Below is the link to the electronic supplementary material.

Supplementary Material 1

## Data Availability

The genome sequence of *Salmonella* phage vB_SalS-SIY1lw was deposited at the National Center for Biotechnology Information (NCBI) database under GenBank accession number # PP105779.1.

## References

[1] Tack, D. M. et al. Preliminary incidence and trends of infections with pathogens transmitted commonly through Food—Foodborne diseases active surveillance Network, 10 U.S. Sites, 2016–2019. *MMWR Morb. Mortal. Wkly. Rep.* **69**, 509–514 (2020). 32352955 10.15585/mmwr.mm6917a1 PMC7206985

[2] Cosby, D. E. et al. *Salmonella* and antimicrobial resistance in broilers: A review. *J. Appl. Poult. Res.* **24**, 408–426 (2015).

[3] McMillan, E. A., Weinroth, M. D. & Frye, J. G. Increased prevalence of *Salmonella* Infantis isolated from Raw chicken and Turkey products in the united States is due to a single clonal lineage carrying the pESI plasmid. *Microorganisms* **10**, 1478 (2022). 35889197 10.3390/microorganisms10071478 PMC9318337

[4] Mejía, L. et al. Genomic epidemiology of *Salmonella* Infantis in ecuador: from poultry farms to human infections. *Front. Vet. Sci.* **7**, 547891 (2020). 33134346 10.3389/fvets.2020.547891 PMC7550756

[5] Montoro-Dasi, L., Lorenzo-Rebenaque, L., Marco-Fuertes, A., Vega, S. & Marin, C. Holistic strategies to control *Salmonella* Infantis: an emerging challenge in the European broiler sector. *Microorganisms* **11**, 1765 (2023). 37512937 10.3390/microorganisms11071765 PMC10386103

[6] Alessiani, A. et al. Occurrence of a new variant of *Salmonella* Infantis lacking somatic antigen. *Microorganisms* **11**, 2274 (2023). 37764118 10.3390/microorganisms11092274 PMC10538023

[7] Piña-Iturbe, A. et al. Genomic characterisation of the population structure and antibiotic resistance of *Salmonella enterica* serovar Infantis in Chile, 2009–2022. *Lancet Reg. Health Am.* **32**, 100711 (2024). 38495315 10.1016/j.lana.2024.100711 PMC10944094

[8] Mattock, J. et al. A one health perspective on *Salmonella enterica* serovar Infantis, an emerging human Multidrug-Resistant pathogen. *Emerg. Infect. Dis.* **30**, 701–710 (2024). 38526070 10.3201/eid3004.231031 PMC10977846

[9] Sevilla-Navarro, S. et al. Fighting *Salmonella* Infantis: bacteriophage-driven cleaning and disinfection strategies for broiler farms. *Front. Microbiol.* **15**, 1401479 (2024). 38812676 10.3389/fmicb.2024.1401479 PMC11134195

[10] Li, C. et al. The spread of pESI-mediated extended-spectrum cephalosporin resistance in *Salmonella* serovars-Infantis, Senftenberg, and Alachua isolated from food animal sources in the United States. *PLoS One*. **19**, e0299354 (2024). 38483966 10.1371/journal.pone.0299354 PMC10939224

[11] O’Sullivan, L., Bolton, D., McAuliffe, O. & Coffey, A. Bacteriophages in food applications: from foe to friend. *Annu. Rev. Food Sci. Technol.* **10**, 151–172 (2019). 30633564 10.1146/annurev-food-032818-121747

[12] Kahn, L. H. et al. From farm management to bacteriophage therapy: strategies to reduce antibiotic use in animal agriculture. *Ann. N Y Acad. Sci.* **1441**, 31–39 (2019). 30924542 10.1111/nyas.14034 PMC6850639

[13] Nobrega, F. L. et al. Targeting mechanisms of tailed bacteriophages. *Nat. Rev. Microbiol.* **16**, 760–773 (2018). 30104690 10.1038/s41579-018-0070-8

[14] Clokie, M. R., Millard, A. D., Letarov, A. V. & Heaphy, S. Phages in nature. *Bacteriophage* **1**, 31–45 (2011). 21687533 10.4161/bact.1.1.14942 PMC3109452

[15] Pelyuntha, W. et al. Isolation and characterization of potential *Salmonella* phages targeting Multidrug-Resistant and major serovars of *Salmonella* derived from broiler production chain in Thailand. *Front. Microbiol.* **12**, 662461 (2021). 34122377 10.3389/fmicb.2021.662461 PMC8195598

[16] Ayala, R. et al. Nearly complete structure of bacteriophage DT57C reveals architecture of head-to-tail interface and lateral tail fibers. *Nat. Commun.* **14**, 8205 (2023). 38081816 10.1038/s41467-023-43824-9 PMC10713586

[17] Turner, D., Kropinski, A. M. & Adriaenssens, E. M. A roadmap for genome-based phage taxonomy. *Viruses*. **13**, 506 (2021). 33803862 10.3390/v13030506 PMC8003253

[18] Huet, A., Duda, R. L., Hendrix, R. W., Boulanger, P. & Conway, J. F. Correct assembly of the bacteriophage T5 procapsid requires both the maturation protease and the portal complex. *J. Mol. Biol.* **428**, 165–181 (2016). 26616586 10.1016/j.jmb.2015.11.019 PMC4738002

[19] Drauch, V., Ibesich, C., Vogl, C., Hess, M. & Hess, C. In-vitro testing of bacteriostatic and bactericidal efficacy of commercial disinfectants against *Salmonella* Infantis reveals substantial differences between products and bacterial strains. *Int. J. Food Microbiol.* **328**, 108660 (2020). 32450393 10.1016/j.ijfoodmicro.2020.108660

[20] Moraes, J. O. et al. Predicting adhesion and biofilm formation boundaries on stainless steel surfaces by five *Salmonella enterica* strains belonging to different serovars as a function of pH, temperature and NaCl concentration. *Int. J. Food Microbiol.* **281**, 90–100 (2018). 29843904 10.1016/j.ijfoodmicro.2018.05.011

[21] Pinto, G., Almeida, C. & Azeredo, J. Bacteriophages to control Shiga toxin-producing *E. coli* - safety and regulatory challenges. *Crit. Rev. Biotechnol.* **40**, 1081–1097 (2020). 32811194 10.1080/07388551.2020.1805719

[22] Vikram, A., Tokman, J. I., Woolston, J. & Sulakvelidze, A. Phage biocontrol improves food safety by significantly reducing the level and prevalence of *Escherichia coli* O157:H7 in various foods. *J. Food Prot.* **83**, 668–676 (2020). 32221572 10.4315/0362-028X.JFP-19-433

[23] Rivera, D. et al. Two phages of the genera felixunavirus subjected to 12 hour challenge on *Salmonella* Infantis showed distinct genotypic and phenotypic changes. *Viruses* **11**, 586 (2019). 31252667 10.3390/v11070586 PMC6669636

[24] Abedon, S. T. Selection for bacteriophage latent period length by bacterial density: A theoretical examination. *Microb. Ecol.* **18**, 79–88 (1989). 24196124 10.1007/BF02030117

[25] Liao, Y. T. et al. A *Tequintavirus* bacteriophage SIA3lw isolated from sewage water with antimicrobial potential against antibiotic-resistant *Salmonella* Infantis. *BMC Microbiol.* (2025). 10.1186/s12866-025-04423-4 PMC12752171 41272491

[26] Skutel, M. et al. T5-like phage BF23 evades host-mediated DNA restriction and methylation. *microLife* **4**, uqad044 (2023). 10.1093/femsml/uqad044 PMC10644984 38025991

[27] Maffei, E. et al. Systematic exploration of *Escherichia coli* phage–host interactions with the BASEL phage collection. *PLoS Biol.* **19**, e3001424 (2021). 34784345 10.1371/journal.pbio.3001424 PMC8594841

[28] Golomidova, A. K. et al. Branched lateral tail fiber organization in T5-Like bacteriophages DT57C and DT571/2 is revealed by genetic and functional analysis. *Viruses* **8**, 26 (2016). 26805872 10.3390/v8010026 PMC4728585

[29] Pedruzzi, I., Rosenbusch, J. P. & Locher, K. P. Inactivation in vitro of the *Escherichia coli* outer membrane protein FhuA by a phage T5-encoded lipoprotein. *FEMS Microbiol. Lett.* **168**, 119–125 (1998). 9812372 10.1111/j.1574-6968.1998.tb13264.x

[30] Golec, P. et al. A role for accessory genes rI.-1 and rI.1 in the regulation of Lysis Inhibition by bacteriophage T4. *Virus Genes*. **41**, 459–468 (2010). 20945083 10.1007/s11262-010-0532-1 PMC2962797

[31] Attrill, E. L., Łapińska, U., Westra, E. R., Harding, S. V. & Pagliara, S. Slow growing bacteria survive bacteriophage in isolation. *ISME Commun.* **3**, 1–9 (2023). 37684358 10.1038/s43705-023-00299-5 PMC10491631

[32] Martinez-Soto, C. E. et al. Multireceptor phage cocktail against *Salmonella enterica* to circumvent phage resistance. *Microlife*. **5**, uqae003 (2024). 38545601 10.1093/femsml/uqae003 PMC10972627

[33] Drauch, V., Mitra, T., Liebhart, D., Hess, M. & Hess, C. Infection dynamics of *Salmonella* Infantis vary considerably between chicken lines. *Avian Pathol.* **51**, 561–573 (2022). 35938538 10.1080/03079457.2022.2108373

[34] Zhang, Y., Chu, M., Liao, Y. T., Salvador, A. & Wu, V. C. H. Characterization of two novel *Salmonella* phages having biocontrol potential against *Salmonella* spp. in gastrointestinal conditions. *Sci. Rep.* **14**, 12294 (2024). 38811648 10.1038/s41598-024-59502-9 PMC11137056

[35] Lennon, M., Liao, Y. T., Salvador, A., Lauzon, C. R. & Wu, V. C. H. Bacteriophages specific to Shiga toxin-producing *Escherichia coli* exist in goat feces and associated environments on an organic produce farm in Northern California, USA. *PLoS One*. **15**, e0234438 (2020). 32525945 10.1371/journal.pone.0234438 PMC7289414

[36] Liao, Y. T. et al. Characterization of polyvalent *Escherichia phage* Sa157lw for the biocontrol potential of *Salmonella* Typhimurium and *Escherichia coli* O157:H7 on contaminated mung bean seeds. *Front. Microbiol.* **13**, 1053583 (2022). 36439834 10.3389/fmicb.2022.1053583 PMC9686305

[37] Liao, Y. T., Zhang, Y., Salvador, A., Harden, L. A. & Wu, V. C. H. Characterization of a T4-like bacteriophage vB_EcoM-Sa45lw as a potential biocontrol agent for Shiga Toxin-Producing *Escherichia coli* O45 contaminated on mung bean seeds. *Microbiol. Spectr.* **10**, e0222021 (2022). 35107386 10.1128/spectrum.02220-21 PMC8809338

[38] Liao, Y. T., Ho, K. J., Zhang, Y., Salvador, A. & Wu, V. C. H. A new Rogue-like *Escherichia* phage UDF157lw to control *Escherichia coli* O157:H7. *Front. Microbiol.* **14**, 1302032 (2024). 38318127 10.3389/fmicb.2023.1302032 PMC10838988

[39] Liao, Y. T. et al. Characterization of a lytic bacteriophage as an antimicrobial agent for biocontrol of Shiga Toxin-Producing *Escherichia coli* O145 strains. *Antibiot. (Basel)*. **8**, 74 (2019). 10.3390/antibiotics8020074 PMC6627115 31195679

[40] Zhang, Y., Liao, Y. T., Salvador, A., Lavenburg, V. M. & Wu, V. C. H. Characterization of two new Shiga Toxin-Producing *Escherichia coli* O103-Infecting phages isolated from an organic farm. *Microorganisms*. **9**, 1527 (2021). 34361962 10.3390/microorganisms9071527 PMC8303462

[41] Antipov, D., Korobeynikov, A., McLean, J. S. & Pevzner, P. A. HybridSPAdes: an algorithm for hybrid assembly of short and long reads. *Bioinformatics*. **32**, 1009–1015 (2016). 26589280 10.1093/bioinformatics/btv688 PMC4907386

[42] Garneau, J. R., Depardieu, F., Fortier, L. C., Bikard, D. & Monot, M. PhageTerm: a tool for fast and accurate determination of phage termini and packaging mechanism using next-generation sequencing data. *Sci. Rep.* **7**, 8292 (2017). 28811656 10.1038/s41598-017-07910-5 PMC5557969

[43] Seemann, T. Prokka: rapid prokaryotic genome annotation. *Bioinformatics*. **30**, 2068–2069 (2014). 24642063 10.1093/bioinformatics/btu153

[44] UniProt Consortium. UniProt: the universal protein knowledgebase in 2021. *Nucleic Acids Res.* **49**, D480–D489 (2021). 33237286 10.1093/nar/gkaa1100 PMC7778908

[45] Lowe, T. M. & Chan, P. P. tRNAscan-SE On-line: integrating search and context for analysis of transfer RNA genes. *Nucleic Acids Res.* **44**, W54–57 (2016). 27174935 10.1093/nar/gkw413 PMC4987944

[46] Malberg Tetzschner, A. M., Johnson, J. R., Johnston, B. D., Lund, O. & Scheutz, F. *Silico* genotyping of *Escherichia coli* isolates for extraintestinal virulence genes by use of Whole-Genome sequencing data. *J. Clin. Microbiol.* **58**, e01269–e01220 (2020). 32669379 10.1128/JCM.01269-20 PMC7512150

[47] Bortolaia, V. et al. ResFinder 4.0 for predictions of phenotypes from genotypes. *J. Antimicrob. Chemother.* **75**, 3491–3500 (2020). 32780112 10.1093/jac/dkaa345 PMC7662176

[48] Zhang, Y., Mao, M., Zhang, R., Liao, Y. T. & Wu, V. C. H. DeepPL: A deep-learning-based tool for the prediction of bacteriophage lifecycle. *PLoS Comput. Biol.* **20**, e1012525 (2024). 39418300 10.1371/journal.pcbi.1012525 PMC11521287

[49] Meier-Kolthoff, J. P. & Göker, M. VICTOR: genome-based phylogeny and classification of prokaryotic viruses. *Bioinformatics*. **33**, 3396–3404 (2017). 29036289 10.1093/bioinformatics/btx440 PMC5860169

[50] Richter, M., Rosselló-Móra, R., Glöckner, O., Peplies, J. & F. & JSpeciesWS: a web server for prokaryotic species circumscription based on pairwise genome comparison. *Bioinformatics*. **32**, 929–931 (2016). 26576653 10.1093/bioinformatics/btv681 PMC5939971

[51] Mahadevan, P., King, J. F., Seto, D. & CGUG. *silico* proteome and genome parsing tool for the determination of ‘core’ and unique genes in the analysis of genomes up to ca. 1.9 Mb. *BMC Res. Notes*. **2**, 168 (2009). 19706165 10.1186/1756-0500-2-168 PMC2738686

[52] Acton, L. et al. Collateral sensitivity increases the efficacy of a rationally designed bacteriophage combination to control *Salmonella enterica*. *J. Virol.* **98**, e01476–e01423 (2024). 38376991 10.1128/jvi.01476-23 PMC10949491

[53] Yamaki, S., Yamazaki, K. & Kawai, Y. Broad host range bacteriophage, EscoHU1, infecting *Escherichia coli* O157:H7 and *Salmonella enterica*: Characterization, comparative genomics, and applications in food safety. *Int. J. Food Microbiol.* **372**, 109680 (2022). 35512432 10.1016/j.ijfoodmicro.2022.109680

[54] Jones, D. T., Taylor, W. R. & Thornton, J. M. The rapid generation of mutation data matrices from protein sequences. *Comput. Appl. Biosci.* **8**, 275–282 (1992). 1633570 10.1093/bioinformatics/8.3.275

[55] Wu, V. C. H. A review of microbial injury and recovery methods in food. *Food Microbiol.* **25**, 735–744 (2008). 18620965 10.1016/j.fm.2008.04.011

