# Supplementary material for: A new Epseptimavirus bacteriophage vB_SalS-SIY1lw as a potential antimicrobial alternative to multidrug-resistant Salmonella Infantis
Source: Sci Rep. 2026 Feb 5;16:5376. doi: 10.1038/s41598-025-31311-8 (PMC12887010; doi:10.1038/s41598-025-31311-8)
Supplement: Supplementary file 1 — Supplementary Material 1 [file 41598_2025_31311_MOESM1_ESM.pdf]

Supplementary materials

Table S1. List of annotated ORFs with location and direction in SIY1lw genome. na means the function is not known.

ORF	Name	Function	Minimum	Maximum	Length	Direction
1	hypothetical protein CDS	na	935	1174	240	reverse
2	hypothetical protein CDS	na	1298	1498	201	reverse
3	DNA-(apurinic or apyrimidinic site) endonuclease CDS	DNA replication/nucleotide metabolism	1495	1839	345	reverse
4	hypothetical protein CDS	na	1839	1946	108	reverse
5	hypothetical protein CDS	na	1946	2158	213	reverse
6	hypothetical protein CDS	na	2212	2442	231	reverse
7	hypothetical protein CDS	na	2551	3045	495	reverse
8	DNA N-6-adenine methyltransferase CDS	DNA replication/nucleotide metabolism	3109	4110	1002	reverse
9	hypothetical protein CDS	na	5202	5420	219	forward
10	hypothetical protein CDS	na	5586	5837	252	forward
11	A2 protein CDS	other function (FST region)	5938	6354	417	forward
12	A1 protein CDS	other function (FST region)	6413	6640	228	forward
13	putative A1 protein CDS	other function (FST region)	6749	8413	1665	forward
14	hypothetical protein CDS	na	8466	8744	279	forward
15	hypothetical protein CDS	na	8777	9169	393	forward
16	deoxynucleoside-5'-monophosphatase CDS	DNA replication/nucleotide metabolism	9257	9991	735	forward

17	hypothetical protein CDS	na	10150	10365	216	reverse
18	hypothetical protein CDS	na	10362	10607	246	reverse
19	hypothetical protein CDS	na	10600	10794	195	reverse
20	tail fiber protein CDS	Structure	10794	10898	105	reverse
21	capsid and scaffold protein CDS	Structure	10971	11285	315	reverse
22	receptor-blocking protein CDS	Host lysis/interaction	11360	11626	267	reverse
23	receptor-binding protein CDS	Structure/capsid proteins	11710	13491	1782	forward
24	terminase small subunit CDS	DNA packaging	13502	13984	483	forward
25	terminase large subunit CDS	DNA packaging	13984	15300	1317	forward
26	nicking site-specific endonuclease CDS	DNA replication/nucleotide metabolism	15457	15852	396	forward
27	portal (Connector) protein CDS	Structure/capsid proteins	15852	17069	1218	forward
28	neck whiskers protein CDS	Structure/capsid proteins	17066	17548	483	forward
29	capsid and scaffold protein CDS	Structure/capsid proteins	17552	18184	633	forward
30	capsid and scaffold protein CDS	Structure/capsid proteins	18202	19578	1377	forward
31	head completion protein CDS	Structure/capsid proteins	19638	20150	513	forward
32	tail completion protein CDS	Structure/capsid proteins	20150	20917	768	forward
33	tail tube terminator protein CDS	Structure/capsid proteins	20921	21406	486	forward
34	major tail protein CDS	Structure/capsid proteins	21433	22842	1410	forward
35	tail fiber protein CDS	Structure/capsid proteins	22847	23746	900	forward
36	putative tape measure chaperone CDS	Structure/capsid proteins	23743	24147	405	forward
37	tape measure chaperone CDS	Structure/capsid proteins	24209	24577	369	forward
38	tail fiber CDS	Structure/capsid proteins	24658	28365	3708	forward
39	distal tail protein CDS	Structure/capsid proteins	28474	29088	615	forward
40	tail protein CDS	Structure/capsid proteins	29085	31934	2850	forward

41	tail fiber CDS	Structure/capsid proteins	31935	33992	2058	forward
42	tail protein CDS	Structure/capsid proteins	33997	34419	423	forward
43	tail fiber protein CDS	Structure/capsid proteins	34419	37772	3354	forward
44	tail fiber protein CDS	Structure/capsid proteins	37815	39902	2088	forward
45	hypothetical protein CDS	na	39940	40152	213	reverse
46	deoxyuridine 5'-triphosphate nucleotidohydrolase CDS	DNA replication/nucleotide metabolism	40169	40615	447	reverse
47	flap endonuclease CDS	DNA replication/nucleotide metabolism	40612	41487	876	reverse
48	D14 protein CDS	DNA replication/nucleotide metabolism	41487	41969	483	reverse
49	recombination-related exonuclease CDS	DNA replication/nucleotide metabolism	41973	43811	1839	reverse
50	recombinase CDS	DNA replication/nucleotide metabolism	43792	44769	978	reverse
51	ssDNA-binding protein CDS	DNA replication/nucleotide metabolism	44809	45582	774	reverse
52	hypothetical protein CDS	na	45575	45937	363	reverse
53	putative ATP-dependent helicase CDS	DNA replication/nucleotide metabolism	46078	47430	1353	reverse
54	DNA-directed DNA polymerase CDS	DNA replication/nucleotide metabolism	47917	50394	2478	reverse
55	DNA primase CDS	DNA replication/nucleotide metabolism	50547	51437	891	reverse
56	SF4 helicase domain-containing protein CDS	DNA replication/nucleotide metabolism	51434	52957	1524	reverse
57	putative transcription factor CDS	other (transcription)	52989	53756	768	reverse
58	NAD-dependent DNA ligase subunit B CDS	DNA replication/nucleotide metabolism	53749	54528	780	reverse
59	DNA ligase (NAD(+)) CDS	DNA replication/nucleotide metabolism	54731	55702	972	reverse

60	hypothetical protein CDS	na	55695	55895	201	reverse
61	transcriptional regulator protein CDS	other (transcription)	55981	56289	309	reverse
62	hypothetical protein CDS	na	56340	56636	297	reverse
63	D3 protein CDS	DNA replication/nucleotide metabolism	56673	57083	411	reverse
64	D2 protein CDS	DNA replication/nucleotide metabolism	57438	58142	705	reverse
65	hypothetical protein CDS	na	58211	58444	234	reverse
66	putative DNA primase CDS	DNA replication/nucleotide metabolism	58428	61313	2886	reverse
67	hypothetical protein CDS	na	61834	62229	396	reverse
68	tail tube protein CDS	Structure/capsid proteins	62239	62667	429	reverse
69	RNA repair protein CDS	DNA replication/nucleotide metabolism	62670	63185	516	reverse
70	hypothetical protein CDS	na	63163	63348	186	reverse
71	hypothetical protein CDS	na	63345	63560	216	reverse
72	NAD-dependent deacetylase of SIR2 family CDS	Host mechanisms	63547	64389	843	reverse
73	hypothetical protein CDS	na	64389	64583	195	reverse
74	hypothetical protein CDS	na	64552	64755	204	reverse
75	hypothetical protein CDS	na	64765	65046	282	reverse
76	ribonucleotide reductase class III (anaerobic) large subunit CDS	DNA replication/nucleotide metabolism	65145	67019	1875	reverse
77	phosphate starvation-inducible protein CDS	Host mechanisms	67611	68363	753	forward
78	hypothetical protein CDS	na	68365	68607	243	forward
79	ribonucleoside-diphosphate reductase CDS	DNA replication/nucleotide metabolism	68624	71059	2436	forward

80	ribonucleoside-diphosphate reductase CDS	DNA replication/nucleotide metabolism	71166	72311	1146	forward
81	dihydrofolate reductase CDS	DNA replication/nucleotide metabolism	72311	72841	531	forward
82	thymidylate synthase CDS	DNA replication/nucleotide metabolism	72838	73692	855	forward
83	proteasome subunit CDS	other	74148	74726	579	forward
84	hypothetical protein CDS	na	74729	74998	270	forward
85	ribonuclease H CDS	DNA replication/nucleotide metabolism	74998	75474	477	forward
86	hypothetical protein CDS	na	75551	75829	279	forward
87	structural protein CDS	Structure/capsid proteins	75913	76428	516	forward
88	tail tape measure protein CDS	Structure/capsid proteins	76490	76705	216	forward
89	hypothetical protein CDS	na	76747	76959	213	forward
90	metallopeptidase CDS	DNA replication/nucleotide metabolism	76990	77691	702	forward
91	hypothetical protein CDS	na	77762	77944	183	forward
92	tail fiber protein CDS	Structure/capsid proteins	78000	78638	639	forward
93	cyclic-phosphate processing receiver domain-containing protein CDS	Host lysis/interaction	79082	79399	318	forward
94	cell wall hydrolase SleB domain-containing protein CDS	Host lysis/interaction	79405	79854	450	forward
95	hypothetical protein CDS	na	79923	80093	171	forward
96	recombination-related exonuclease CDS	DNA replication/nucleotide metabolism	80093	80536	444	forward
97	tRNA-Arg	tRNA	81408	81482	75	forward
98	band 7 domain-containing protein CDS	Structure/capsid proteins	81721	82665	945	forward
99	hypothetical protein CDS	na	82983	83411	429	reverse

100	hypothetical protein CDS	na	83434	84420	987	reverse
101	trifunctional NAD biosynthesis/regulator proteinNadR CDS	DNA replication/nucleotide metabolism	84686	85741	1056	forward
102	nicotinamide mononucleotide transporter CDS	DNA replication/nucleotide metabolism	85743	86420	678	forward
103	hypothetical protein CDS	na	86527	86823	297	forward
104	tRNA-Ser	tRNA	86875	86968	94	forward
105	tRNA-Met	tRNA	86974	87051	78	forward
106	hypothetical protein CDS	na	87069	87254	186	forward
107	hypothetical protein CDS	na	87256	87594	339	forward
108	tRNA-Leu	tRNA	87603	87679	77	forward
109	hypothetical protein CDS	na	87695	87817	123	forward
110	hypothetical protein CDS	na	87831	88037	207	forward
111	hypothetical protein CDS	na	88128	88403	276	forward
112	tRNA-Tyr	tRNA	88594	88684	91	forward
113	tRNA-Glu	tRNA	88692	88768	77	forward
114	tRNA-Trp	tRNA	88777	88853	77	forward
115	homing endonuclease CDS	DNA replication/nucleotide metabolism	88952	89224	273	forward
116	hypothetical protein CDS	na	89275	89499	225	forward
117	tRNA-Cys	tRNA	89674	89749	76	forward
118	tRNA-Asn	tRNA	89756	89841	86	forward
119	hypothetical protein CDS	na	89869	90054	186	forward
120	tRNA-Asp	tRNA	90064	90140	77	forward
121	tRNA-Lys	tRNA	90217	90292	76	forward
122	tRNA-Pro	tRNA	91213	91289	77	forward
123	tRNA-Met	tRNA	91296	91373	78	forward
124	hypothetical protein CDS	na	91395	91559	165	forward
125	tRNA-Lys	tRNA	91562	91640	79	forward
126	hypothetical protein CDS	na	91658	91975	318	forward
127	tRNA-Val	tRNA	92109	92182	74	forward

128	hypothetical protein CDS	na	92208	92561	354	forward
129	tRNA-Ala	tRNA	92823	92901	79	forward
130	tRNA-Ala	tRNA	92907	92982	76	forward
131	ABC transporter permease CDS	other	93002	93220	219	forward
132	tRNA-Leu	tRNA	93228	93308	81	forward
133	tRNA-Ser	tRNA	93483	93574	92	forward
134	tRNA-Ser	tRNA	93582	93671	90	forward
135	hypothetical protein CDS	na	93690	93893	204	forward
136	tRNA-His	tRNA	94255	94331	77	forward
137	tRNA-Arg	tRNA	94338	94412	75	forward
138	hypothetical protein CDS	na	94437	94637	201	forward
139	tRNA-Gln	tRNA	94645	94720	76	forward
140	tRNA-Gln	tRNA	94727	94802	76	forward
141	tRNA-Gly	tRNA	94809	94883	75	forward
142	hypothetical protein CDS	na	94900	95121	222	forward
143	hypothetical protein CDS	na	95114	95278	165	forward
144	tRNA-Thr	tRNA	95289	95363	75	forward
145	hypothetical protein CDS	na	95438	95731	294	forward
146	tRNA-Ile	tRNA	95733	95808	76	forward
147	2-ketobutyrate formate-lyase CDS	na	96260	96628	369	forward
148	hypothetical protein CDS	na	96848	97003	156	forward
149	hypothetical protein CDS	na	97068	97253	186	forward
150	hypothetical protein CDS	na	97312	97659	348	forward
151	hypothetical protein CDS	na	97737	98018	282	forward
152	hypothetical protein CDS	na	98011	98226	216	forward
153	hypothetical protein CDS	na	98219	98518	300	forward
154	hypothetical protein CDS	na	98511	98930	420	forward
155	putative membrane protein CDS	Structural proteins	99177	99461	285	forward
156	putative membrane protein CDS	Structural proteins	99578	99925	348	forward

157	hypothetical protein CDS	na	100071	100769	699	forward
158	I-spanin CDS	Host lysis/interaction	100726	101175	450	forward
159	DNMP kinase CDS	Host lysis/interaction	101459	102211	753	forward
160	ATP-dependent Clp protease proteolytic subunit CDS	Host lysis/interaction	102224	102823	600	forward
161	holin CDS	Host lysis/interaction	103112	103636	525	forward
162	endolysin CDS	Host lysis/interaction	103633	104046	414	forward
163	hypothetical protein CDS	na	104614	105024	411	forward
164	thioredoxin CDS	DNA replication/nucleotide metabolism	105017	105307	291	forward
165	hypothetical protein CDS	na	105469	105789	321	forward
166	putative serine/threonine protein phosphatase CDS	Other	105789	106652	864	forward
167	hypothetical protein CDS	na	106652	107020	369	forward
168	lipoprotein CDS	Structural protein	107020	107226	207	forward
169	phosphoesterase CDS	DNA replication/nucleotide metabolism	107226	107810	585	forward
170	hypothetical protein CDS	na	107803	107904	102	forward
171	hypothetical protein CDS	na	107972	108403	432	forward
172	hypothetical protein CDS	na	108482	108733	252	forward
173	hypothetical protein CDS	na	108733	109140	408	forward
174	putative membrane protein CDS	Structure	109137	109418	282	forward
175	putative membrane protein CDS	Structure	109415	109660	246	forward
176	hypothetical protein CDS	na	109650	109982	333	forward
177	hypothetical protein CDS	na	110083	110283	201	forward
178	hypothetical protein CDS	na	110280	110747	468	forward
179	capsid and scaffold protein CDS	Structure	110689	111072	384	forward
180	hypothetical protein CDS	na	111117	111647	531	forward

181	hypothetical protein CDS	na	111747	112358	612	forward
182	hypothetical protein CDS	na	112358	113248	891	forward
183	hypothetical protein CDS	na	114766	115005	240	reverse
184	hypothetical protein CDS	na	115129	115329	201	reverse
185	DNA-(apurinic or apyrimidinic site) endonuclease CDS	DNA replication/nucleotide metabolism	115326	115670	345	reverse
186	hypothetical protein CDS	na	115670	115777	108	reverse
187	hypothetical protein CDS	na	115777	115989	213	reverse
188	hypothetical protein CDS	na	116043	116273	231	reverse
189	hypothetical protein CDS	na	116382	116876	495	reverse
190	DNA N-6-adenine methyltransferase CDS	DNA replication/nucleotide metabolism	116940	117941	1002	reverse
191	hypothetical protein CDS	na	119033	119251	219	forward
192	hypothetical protein CDS	na	119417	119668	252	forward
193	A2 protein CDS	other function (FST region)	119769	120185	417	forward
194	A1 protein CDS	other function (FST region)	120244	120471	228	forward
195	putative A1 protein CDS	other function (FST region)	120580	122244	1665	forward
196	hypothetical protein CDS	na	122297	122575	279	forward
197	hypothetical protein CDS	na	122608	123000	393	forward
198	deoxynucleoside-5'-monophosphatase CDS	DNA replication/nucleotide metabolism	123088	123822	735	forward

Table S2. Information of the bacterial strains used in this study.

Strains	Strain Ref. No.	Source
Salmonella	*Salmonella* Infantis (BAA-1675)	ATCC
	Salmonella Infantis (RM2480)	CDC (human)
	Salmonella Infantis (RM2481)	USDA-ARS-PSM
	Salmonella Infantis (RM19091)	USDA-ARS-PSM
	Salmonella Infantis (RM19096)	USDA-ARS-PSM
	Salmonella Infantis (FSIS9799)	Product-Raw-Intact-Chicken by the USDA FSIS
	Salmonella Infantis (FSIS9916)	Product-Raw-Intact-Chicken by the USDA FSIS
	Salmonella Infantis (FSIS4897)	Young Chicken Carcass Rinse by the USDA FSIS
	Salmonella Infantis (FSIS4900)	Comminuted or Otherwise Nonintact-Chicken by the USDA FSIS
	Salmonella Infantis (FSIS4921)	Comminuted or Otherwise Nonintact-Beef by the USDA FSIS
	Salmonella Infantis (FSIS9851)	Comminuted or Otherwise Nonintact-Chicken by the USDA FSIS
	Salmonella Infantis (FSIS9861)	Comminuted or Otherwise Nonintact-Chicken by the USDA FSIS
	Salmonella Infantis (FSIS7821)	Comminuted or Otherwise Nonintact-Chicken by the USDA FSIS
	Salmonella Infantis (FSIS5221)	Comminuted or Otherwise Nonintact-Turkey by the USDA FSIS
	Salmonella Infantis (FSIS7823)	Young Chicken Carcass Rinse by the USDA FSIS
Other *Salmonella* serovars	*Salmonella* Typhimurium 14028	ATCC
	Salmonella Montevideo 51	USDA-ARS-PSM
	Salmonella Newport H1073	USDA-ARS-PSM
	Salmonella Heidelberg 45955	USDA-ARS-PSM
	Salmonella Enteritidis PT-30	USDA-ARS-PSM

Shiga toxin-producing *E. coli*	O26:H11 (BAA-2196)	stool
	O26:H11 (SJ2)	USDA-ARS-ERCC
	O45:H- (RM10729)	cattle
	O103:H2 (RM10744)	cattle feces
	O111:H- (RM11765)	water
	O121:H19 (96-1585)	CDC
	O121:H- (RM8082)	cattle feces
	O145:H28 (RM13514)	clinical isolate, linked to the 2010 multi-state outbreak of *E. coli* O145 infection associated with consumption of contaminated romaine lettuce in US
	E. coli O157:H7 (ATCC 35150)	ATCC
	E. coli O157:H7 (ATCC 43888)	ATCC
E. albertii	*E. albertii* RM9973	American crow
	E. albertii RM9974	American crow
	E. albertii RM15113	Oregon Junco
Non-pathogenic *E. coli*	ATCC 13706	ATCC
	ATCC 15597	ATCC
	TVS 353	irrigation water (Salinas, CA)

Table S3. Antibiotic susceptibility test for *S.* Infantis (FSIS7823) and *S.* Infantis (FSIS4921) used in this study. (S) means bacterium is sensitive to the drug. (R) means bacterium is resistant to the drug. (I) means the bacterium is sensitive to the drug at certain dosages. Susceptible Dose-Dependent (SDD) means the bacterial susceptibility is dosage dependent.

**Antibiotics**	**Resistance profiles**	
	S. Infantis FSIS7823	*S.* Infantis FSIS4921
Moxifloxacin (5 μg)	S	S
Ciprofloxacin (5 μg)	I	I
Ceftazidimine (30 μg)	S	S
Cefoperazone (75 μg)	R	S
Cefoxitin (30 μg)	S	S
Cefotaxime (30 μg)	R	I
Ceftriaxone (30 μg)	S	S
Cefepime (30 μg)	SDD	S
Cefazolin (30 μg)	R	S
Streptomycin (10 μg)	R	I
Amikacin (30 μg)	S	S
Kanamycin (30 μg)	S	R
Gentamicin (10 μg)	R	R
Meropenem (10 μg)	S	S
Imipenem (10 μg)	S	S
Piperacillin (100 μg)	R	S
Ampicillin (10 μg)	R	S
Amoxicillin-clavulanate (30 μg)	R	S
Ampicillin-sulbactam (20 μg)	S	S
Tetracycline (30 μg)	I	R
Chloramphenicol (30 μg)	R	R
Trimethoprim-sulfamethoxazole (25 μg)	S	S

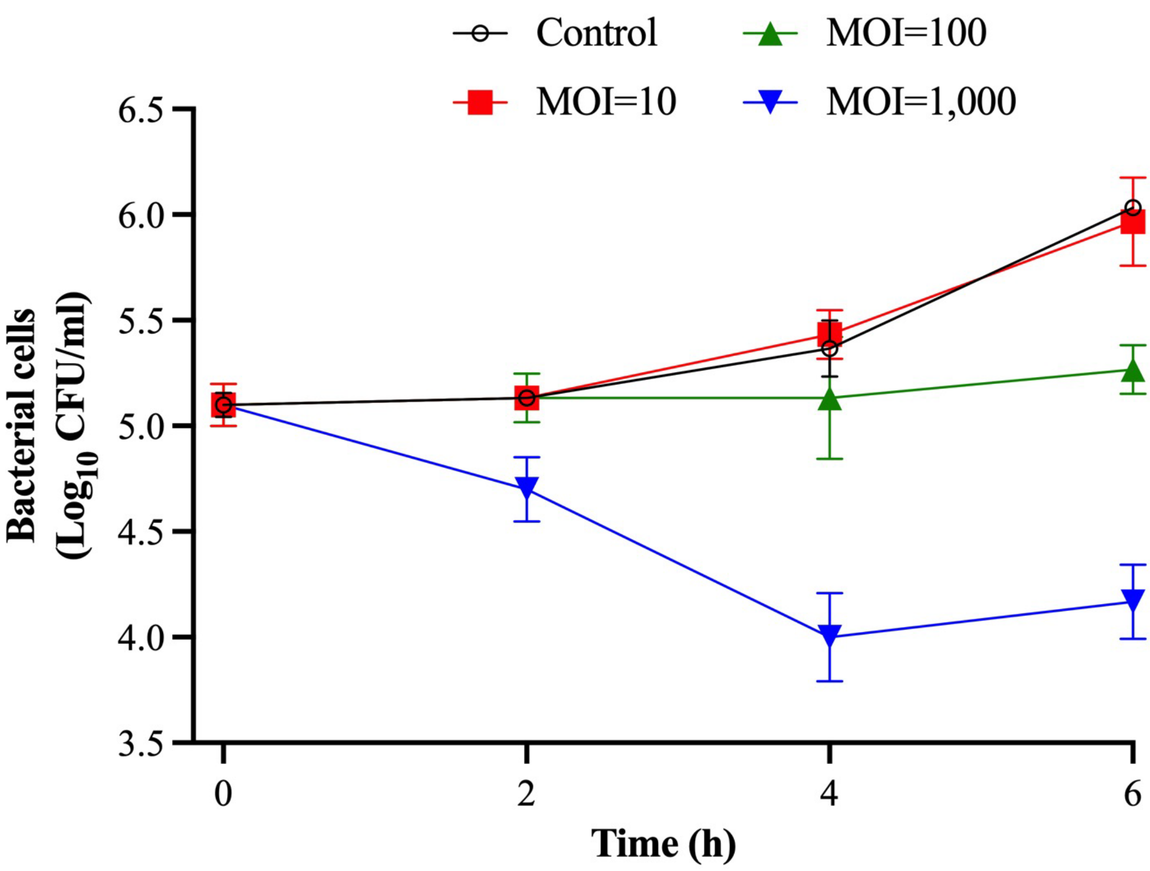

Figure S1. The antimicrobial activity of SIY1lw with various MOIs (10, 100, and 1,000) against *Salmonella* Newport and in LB broth at 25°C for 6 hours. The control group only contains bacterial culture. The error bars show the standard error of the mean (SEM).

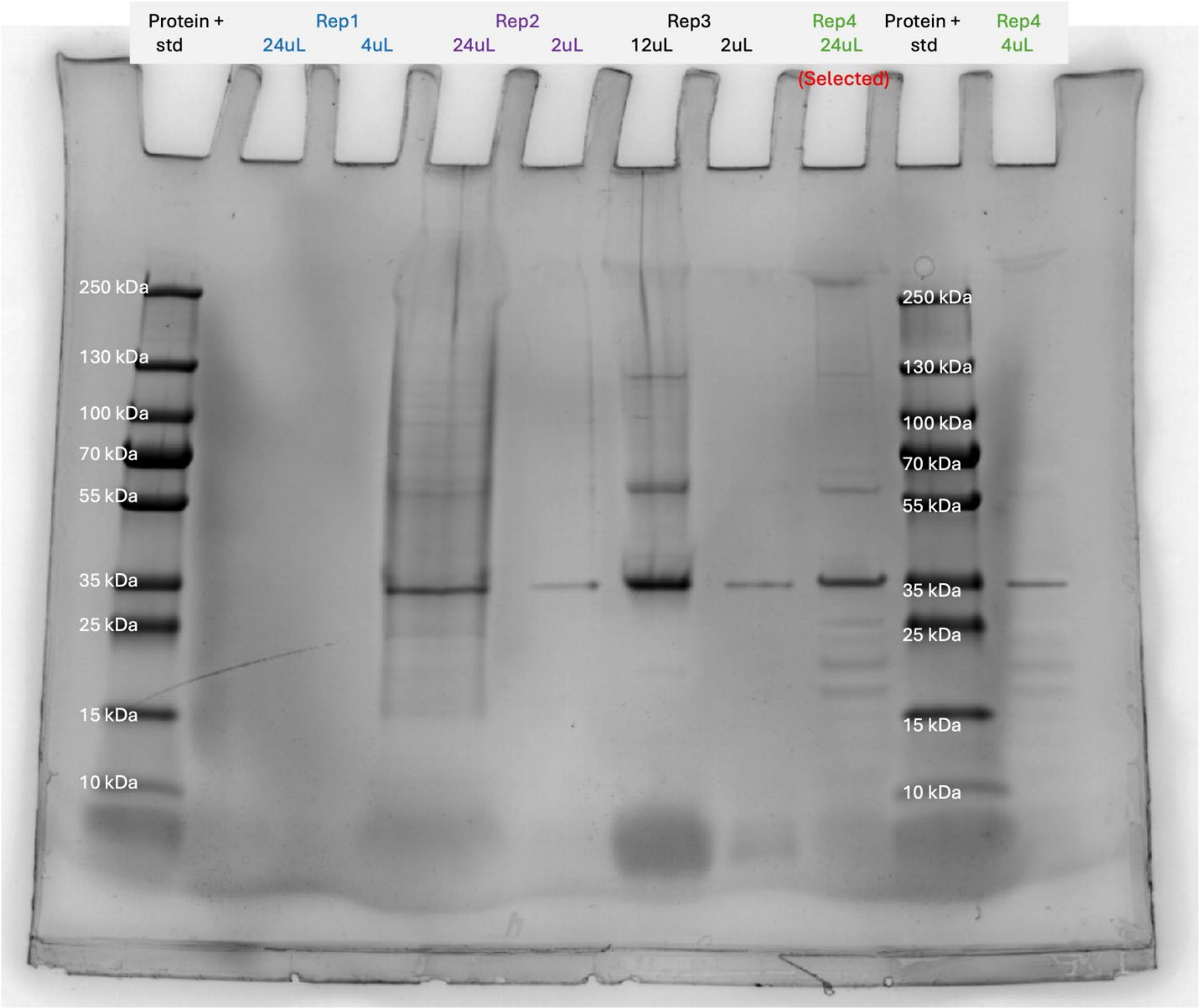

Figure S2. Uncropped sodium dodecyl sulfate-polyacrylamide gel (SDS-PAGE) image for phage SIY1lw proteins. The CsCl-purified phage SIY1lw was added with Laemmli buffer before loading the samples from rep1 (24 and 4 μL), rep2 (24 and 2 μL), rep3 (12 and 2 μL), and rep4 (24 and 4 μL) into the wells from left to right of an SDS-PAGE. The lane loaded with 24 μL from rep4 was used in Fig. 8 of the manuscript.
